# TIC-FusionNet: A multimodal deep learning framework with temporal decomposition and attention-based fusion for time series forecasting

**DOI:** 10.1371/journal.pone.0333379

**Published:** 2025-10-09

**Authors:** Liyu Chen, Xiangwei Fan

**Affiliations:** 1 International Business School, Qingdao Huanghai University, Qingdao, China; 2 Qingdao Talent and Enterprise Service Group Co., Ltd., Qingdao, China; Chitkara University Institute of Engineering and Technology, INDIA

## Abstract

We propose **TIC-FusionNet**, a trend-aware multimodal deep learning framework for time series forecasting with integrated visual signal analysis, aimed at addressing the limitations of unimodal and short-range dependency models in noisy financial environments. The architecture combines Exponential Moving Average (EMA) decomposition for denoising and trend extraction, a lightweight Linear Transformer for efficient long-sequence temporal modeling, and a spatial–channel CNN with CBAM attention to capture morphological patterns from candlestick chart images. A gated fusion mechanism adaptively integrates numerical and visual modalities based on context relevance, enabling dynamic feature weighting under varying market conditions. We evaluate TIC-FusionNet on six real-world stock datasets, including four major Chinese and U.S. companies—Amazon, Tesla, Kweichow Moutai, Ping An Insurance, China Vanke—and Apple—covering diverse market sectors and volatility patterns. The model is compared against a broad range of baselines, including statistical models (ARIMA), classical machine learning methods (Random Forest, SVR), recurrent and convolutional neural networks (LSTM, TCN, CNN-only), and recent Transformer-based architectures (Informer, Autoformer, Crossformer, iTransformer). Experimental results demonstrate that TIC-FusionNet achieves consistently superior predictive accuracy and generalization, outperforming state-of-the-art baselines across all datasets. Extensive ablation studies verify the critical role of each architectural component, while attention-based interpretability analysis highlights the dominant technical indicators under different volatility regimes. These findings not only confirm the effectiveness of multimodal integration in capturing complementary temporal–visual cues, but also provide valuable insights into model decision-making. The proposed framework offers a robust, scalable, and interpretable solution for multimodal temporal prediction tasks, with strong potential for deployment in intelligent forecasting, sensor fusion, and risk-aware decision-making systems.

## 1 Introduction

Multivariate time series forecasting under nonlinear, high-noise, and nonstationary conditions remains a core challenge across domains such as engineering systems, environmental monitoring, and computational finance [35]. In the financial context, stock prices exhibit complex temporal dependencies and modality heterogeneity, driven by a combination of technical signals, macroeconomic indicators, market sentiment, and latent structural events [19,20]. These dynamic influences often interact in nonlinear and high-dimensional ways, complicating traditional modeling paradigms.

Conventional statistical models such as ARIMA [21] and GARCH [1] exhibit limited robustness when confronted with abrupt trend shifts, regime changes, or structural breaks inherent in financial time series. While classical machine learning approaches such as Support Vector Machines (SVM) [4] and Random Forests (RF) [5] enhance the ability to capture nonlinear relationships, they remain heavily reliant on manual feature engineering and often struggle to model long-term temporal dependencies or to exploit interactions between heterogeneous modalities [37].

Deep learning methods have been extensively explored to overcome these limitations. Recurrent Neural Networks (RNNs) and their variants such as Long Short-Term Memory (LSTM) and Gated Recurrent Units (GRU) [7] are capable of modeling sequential dependencies, yet they often suffer from vanishing or exploding gradients and exhibit scalability constraints when handling very long sequences. Convolutional Neural Networks (CNNs) [22] effectively capture local temporal patterns, whereas recent Transformer-based architectures—including Autoformer [14], Crossformer [13], and iTransformer [12]—have further advanced long-sequence modeling by leveraging self-attention mechanisms, enabling multi-scale temporal dependency capture and improved representation learning in complex time series scenarios [38]. Nevertheless, the majority of these approaches are restricted to structured numerical inputs and overlook auxiliary modalities such as candlestick chart images, which encapsulate rich morphological cues pertinent to identifying temporal patterns, trend reversals, and volatility regimes [39].

In response, we propose **TIC-FusionNet**, a novel multimodal deep learning framework designed to integrate structured time series and visual signals for robust and interpretable forecasting. The model introduces an Exponential Moving Average (EMA) decomposition module to isolate long-term trend dynamics [13], a dual-branch encoder comprising a computationally efficient Linear Transformer for high-dimensional time series modeling—enabling long-range temporal pattern capture with reduced complexity—and a CNN with CBAM [23] for visual chart analysis. These modality-specific embeddings are subsequently integrated via a gated fusion mechanism [24,36], allowing adaptive cross-modal feature alignment and dynamic weighting based on contextual volatility.

We evaluate TIC-FusionNet on six representative real-world stock datasets spanning distinct markets (U.S. and China) and diverse industry sectors, including technology, consumer goods, and financial services. The U.S. market is represented by Amazon, Tesla, and Apple, while the Chinese market includes Kweichow Moutai, Ping An Insurance, and China Vanke. Empirical results demonstrate that TIC-FusionNet delivers consistent and substantial improvements over a comprehensive set of baselines, including traditional statistical methods (ARIMA), recurrent models (LSTM), convolutional architectures, and recent Transformer-based state-of-the-art approaches such as Informer, Autoformer, Crossformer, and iTransformer. Across multiple evaluation metrics (RMSE, MAE, MAPE, SMAPE, *R*^2^), the proposed framework exhibits superior generalization capability and robustness under varying market conditions. Furthermore, extensive ablation studies confirm the contribution of each architectural module and highlight the synergistic benefits of multimodal representation learning and trend-aware modeling [16].

The contributions of this paper are as follows:

- We propose TIC-FusionNet, a trend-aware multimodal neural architecture integrating time series decomposition and image-based technical feature modeling under a unified fusion framework.
- We incorporate a channel-spatial attention mechanism and gated fusion strategy for dynamic modality weighting, enhancing predictive accuracy and interpretability.
- We conduct extensive experiments and ablation studies on diverse datasets to demonstrate the model’s robustness, cross-market adaptability, and component effectiveness.

## 2 Related work

### 2.1 Traditional methods for financial time series forecasting

Financial time series prediction has traditionally relied on statistical models such as AutoRegressive Integrated Moving Average (ARIMA) and Generalized AutoRegressive Conditional Heteroskedasticity (GARCH) [1,21]. ARIMA captures linear dependencies and seasonality through autoregressive and moving average components, but assumes data stationarity and often fails to accommodate nonlinear shifts. GARCH models extend this capability by modeling time-varying volatility and capturing volatility clustering—a common phenomenon in financial markets [2]. However, both families of models are fundamentally limited in modeling nonlinear dependencies and adapting to structural breaks and sudden regime transitions [3]. Moreover, their forecasting performance degrades significantly under noisy, high-dimensional, and nonstationary data conditions [37,40].

To overcome these limitations, classical machine learning methods such as Support Vector Machines (SVM) [4], Random Forests (RF) [5], and Gradient Boosting Trees (GBT) [6] have been applied in financial forecasting. These approaches provide greater flexibility in handling nonlinearity and complex interactions. SVMs leverage kernel functions to learn nonlinear relationships in feature space [26], while RF and GBT use ensemble learning to improve generalization [27]. Nonetheless, these models still require extensive manual feature engineering, are sensitive to input representation, and lack native mechanisms for temporal modeling [28]. Their inability to capture long-range dependencies and sequential dynamics limits their effectiveness in modeling volatile, multivariate time series characteristic of financial data.

### 2.2 Deep learning-based forecasting models

Deep learning models have demonstrated superior capability in capturing nonlinearity, high-dimensional dependencies, and long-range temporal structures. Recurrent Neural Networks (RNNs) [30,31], especially Long Short-Term Memory (LSTM) and Gated Recurrent Unit (GRU) architectures [29], have been widely adopted for financial forecasting [7,41]. These models leverage gating mechanisms to mitigate vanishing gradient issues and retain long-term contextual information [32]. Numerous studies have shown their effectiveness in modeling stock price dynamics, trading signals, and multi-asset relationships [33,42]. However, RNN-based models suffer from high computational cost and poor parallelization, making them less scalable to large datasets or long sequences [9].

To enhance representational capacity, hybrid architectures that combine Convolutional Neural Networks (CNNs) and LSTMs have emerged [22,43]. CNNs are adept at extracting local spatial patterns and short-term signals, particularly when financial data is visualized as images or matrices (e.g., candlestick charts, spectrograms). CNN-LSTM hybrids first identify structural patterns through convolutional filters and subsequently model temporal evolution via LSTM units [8]. However, such architectures process spatial and temporal features sequentially and often lack mechanisms for joint representation learning across modalities [44].

Transformer-based models [9] have recently reshaped time series modeling due to their self-attention mechanism, enabling global dependency modeling without recurrence. Informer [10] improves efficiency by applying ProbSparse attention to focus on dominant queries, while Autoformer [14] incorporates a series decomposition block to separate trend and seasonal components for more stable long-term forecasting. Crossformer [13] explicitly models cross-dimension dependencies, capturing both intra- and inter-variable correlations in multivariate time series. iTransformer [12] introduces instance-wise attention for better generalization across heterogeneous time series. These architectures have achieved state-of-the-art performance in long-sequence forecasting tasks and are increasingly adopted in high-frequency trading, volatility prediction, and multi-asset modeling. However, most of them still operate solely on structured numerical inputs and do not leverage cross-modality correlations—such as visual signals from technical charts—thereby limiting robustness and generalization under complex and noisy market scenarios.

### 2.3 Multimodal learning in financial time series forecasting

In response to the increasing complexity and multimodality of financial environments, recent research has focused on integrating heterogeneous data sources—such as technical indicators, macroeconomic variables, news sentiment, and candlestick chart images—into unified multimodal frameworks [18]. Multimodal learning enables models to extract complementary information across input modalities, improving resilience to missing data, capturing richer representations, and enhancing forecasting accuracy [45].

For instance, He and Gu [16] proposed a Multi-modal Attention Network (MAN) that fuses stock price series with sentiment signals from social media using attention weighting. Zong et al. [25] developed a gated cross-attention network (MSGCA) that integrates numerical prices, financial news, and macro indicators. Their architecture effectively learns cross-modality relevance, improving generalization across diverse market conditions. In the visual domain, Khodaee et al. [15] designed a CNN-LSTM-ResNet architecture that processes 2D K-line chart segments, enhancing turning point prediction by capturing morphological structures in price evolution.

Despite these advances, many existing frameworks adopt late fusion or treat each modality independently [16], failing to capture complex cross-modal interactions or dynamic saliency across modalities. Moreover, few models explicitly account for trend decomposition or noise suppression prior to fusion [14]. To address these gaps, we propose **TIC-FusionNet**, a unified multimodal forecasting model that integrates an Exponential Moving Average (EMA) decomposition module [13], a dual-branch encoder consisting of a Linear Transformer and CNN+CBAM pipeline [12,23], and a gated fusion mechanism [24,34]. This architecture supports fine-grained feature extraction, trend-aware forecasting, and volatility-sensitive attention across both numerical and visual modalities [17]. Experimental results confirm that TIC-FusionNet offers improved robustness, accuracy, and interpretability under diverse market conditions and cross-market settings [18].

## 3 Proposed framework

This section presents the overall architecture of TIC-FusionNet, its operational flow, and experimental configuration, addressing both the design and deployment perspectives.

### 3.1 Overall architecture

The proposed multimodal stock prediction framework, TIC-FusionNet, integrates complementary information from numerical and visual modalities through three core modules (Fig 3). (1) A time-series branch employs Exponential Moving Average (EMA) decomposition and a Linear Transformer to capture both short-term dependencies and long-term trend structures. (2) An image branch processes candlestick charts with technical indicators via a spatial-channel CNN enhanced with a Convolutional Block Attention Module (CBAM), highlighting salient visual patterns. (3) A gated fusion module adaptively combines the latent features from both branches, dynamically adjusting their contributions based on market conditions. This design allows TIC-FusionNet to produce robust and accurate 5-day log-return forecasts, outperforming conventional unimodal or static-fusion approaches.

### 3.2 Flow of operations

**Fig 1:**
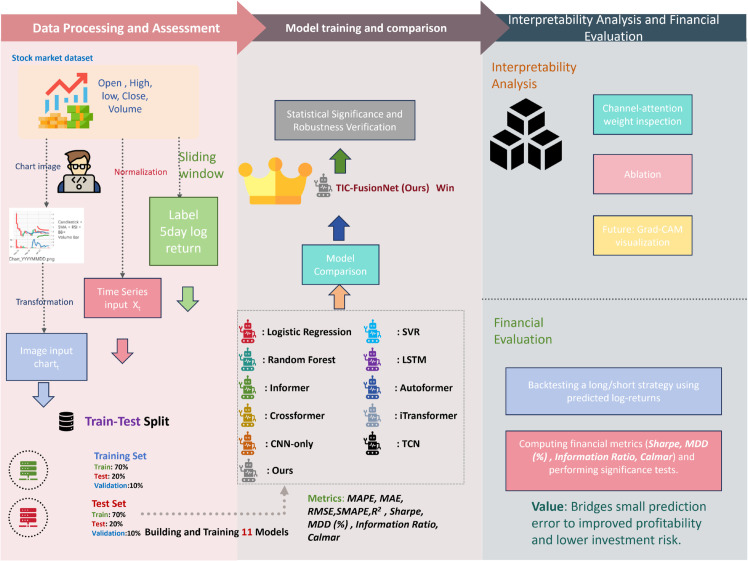
Overall operational flow of TIC-FusionNet, including data preprocessing, model training and benchmarking, interpretability inspection, and financial evaluation.

Fig 1 overviews the end-to-end pipeline of **TIC-FusionNet** across three stages: (i) *Data Processing and Assessment*, (ii) *Feature Analysis and Model Training*, and (iii) *Interpretability and Financial Evaluation*. In Stage (i), historical OHLCV series and technical indicators are aligned on native trading calendars, cleaned, normalized, and rendered into standardized candlestick charts with indicator overlays; labels are defined as 5-day log-returns with strict chronological splits to avoid leakage. In Stage (ii), the temporal branch applies EMA-based trend decomposition and Linear Transformer encoding, while the image branch uses an SC-CNN with CBAM to extract spatial–channel patterns; a gated fusion module adaptively combines modality-specific embeddings, and models are trained under a unified protocol (Adam, MSE, early stopping, ReduceLROnPlateau). In Stage (iii), we examine channel/spatial attention for interpretability, and evaluate real-world utility via a simple long/short backtest, reporting Sharpe, Information Ratio, Calmar ratio, and Maximum Drawdown, with statistical significance tested against baselines.

Fig 1 illustrates the complete workflow of **TIC-FusionNet** in three sequential stages: *Data Processing and Assessment*, *Model Training and Comparison*, and *Interpretability and Financial Evaluation*.

- **Stage 1 – Data Processing and Assessment:** Historical OHLCV (Open, High, Low, Close, Volume) data from multiple stocks are first normalized and aligned. Candlestick chart images with overlaid technical indicators (e.g., RSI, moving averages) are generated in parallel with numerical time series. A sliding window approach is applied to create input sequences (*X*_*t*_) and corresponding 5-day log-return labels. The dataset is split into training, validation, and test subsets with a 70%–10%–20% ratio, ensuring chronological integrity.
- **Stage 2 – Model Training and Comparison:** Both numerical and visual inputs are fed into modality-specific encoders (EMA+Linear Transformer for numerical data, SC-CNN+CBAM for image data), whose outputs are fused via a gated mechanism. The proposed TIC-FusionNet is benchmarked against ten baseline models, including classical machine learning methods (LR, SVR, RF), recurrent/temporal networks (LSTM, TCN), and recent SOTA transformers (Informer, Autoformer, Crossformer, iTransformer), as well as CNN-only and numerical-only variants. Evaluation metrics include MAPE, MAE, RMSE, SMAPE, *R*^2^, Sharpe ratio, MDD (%), Information Ratio, and Calmar ratio. Statistical significance and robustness verification are performed.
- **Stage 3 – Interpretability and Financial Evaluation:** Model interpretability is achieved via channel-attention weight inspection, ablation analysis, and prospective Grad-CAM visualization of candlestick patterns. Financial evaluation is conducted by backtesting a simple long/short strategy on predicted log-returns, computing key metrics (Sharpe, MDD, Information Ratio, Calmar), and linking reduced prediction error to improved profitability and reduced investment risk.

### 3.3 Dataset description

To comprehensively evaluate the performance and generalization ability of the proposed **TIC-FusionNet** framework, we conduct experiments on six real-world stock datasets covering multiple industries, market conditions, and geographical regions. The selected assets include two from the U.S. technology sector (Amazon and Apple), one from the U.S. automotive and clean energy sector (Tesla), and three from representative sectors in the Chinese market (Kweichow Moutai, Ping An Insurance, and China Vanke). This diversified selection spans different volatility regimes, trading behaviors, and macroeconomic contexts, thereby enabling a rigorous assessment of the proposed model’s multimodal forecasting capability and cross-market adaptability.

All datasets were retrieved in CSV format from reputable financial data providers (Yahoo Finance, https://finance.yahoo.com/; Investing.com, https://www.investing.com/) and underwent consistent preprocessing, including (i) missing value imputation, (ii) adjustment for stock splits and dividends, and (iii) alignment to valid trading days.

The datasets are publicly accessible via the URLs listed below.

- **Amazon (U.S. Technology Sector)**— **6,155 daily records** from *May 15, 1997 to October 27, 2021*, covering multiple financial cycles including the dot-com bubble and the COVID-19 pandemic. Attributes: date, open, high, low, close, adjusted close, and volume. Source: Yahoo Finance (https://finance.yahoo.com/) and Investing.com (https://cn.investing.com/equities/amazon-com-inc).
- **Apple (U.S. Technology Sector)**— **10,332 daily records** from *December 12, 1980 to September 30, 2022*, spanning over four decades of corporate growth and global market expansion. Attributes: date, open, high, low, close, adjusted close, and volume. Source: Yahoo Finance (https://finance.yahoo.com/) and Investing.com (https://cn.investing.com/equities/apple-computer-inc).
- **Tesla (U.S. Automotive and Clean Energy Sector)**— **3,051 daily records** from *June 29, 2010 to September 30, 2022*, characterized by high volatility and strong sensitivity to both automotive industry and renewable energy policy shifts. Attributes: date, open, high, low, close, adjusted close, and volume. Source: Investing.com (https://cn.investing.com/equities/tesla-motors-historical-data).
- **Kweichow Moutai (China Consumer Sector)**— **1,723 daily records** from *September 2, 2015 to September 30, 2022*, representing the Chinese high-end liquor sector. Includes standard OHLCV features plus *open interest (OpenInt)*. Source: Investing.com (https://cn.investing.com/equities/moutai).
- **Ping An Insurance (China Financial Sector)**— **1,723 daily records** from *September 2, 2015 to September 30, 2022*, covering one of China’s largest insurance and financial services companies. Attributes match those of Moutai. Source: Investing.com (https://cn.investing.com/equities/ping-an).
- **China Vanke (China Real Estate Sector)**— **1,588 daily records** from *September 2, 2015 to September 30, 2022*, reflecting housing market cycles and policy-driven fluctuations. Attributes match those of Moutai. Source: Investing.com (https://cn.investing.com/equities/vanke-a).

Together, these six datasets constitute a highly diversified cross-market and cross-sector portfolio—spanning technology, automotive and clean energy, consumer goods, financial services, and real estate across both U.S. and Chinese markets. This breadth ensures that the evaluation encompasses heterogeneous market conditions, including stable growth phases, high-volatility speculative environments, policy-driven fluctuations, and industry-specific shocks. Furthermore, the temporal coverage of these datasets includes multiple economic cycles and both pre- and post-pandemic periods, providing a rigorous benchmark for assessing model robustness and generalization under extreme and rapidly shifting market scenarios.

To further assess robustness against varying historical data lengths, we perform supplementary experiments (reported in Supporting Information S1 Appendix) where Chinese stock datasets are resampled to 6,155 trading days and the foreign stock datasets (Amazon, Apple, Tesla) are trimmed to 1,500 trading days. This allows us to evaluate TIC-FusionNet’s stability under both extended and shortened input horizons.

### 3.4 Experimental setup

To ensure objective and reproducible performance comparisons, all models were implemented and evaluated within a standardized computational setup. Experiments were executed on a single NVIDIA Tesla V100 GPU with 32GB memory in FP32 precision, using Python 3.8 and PyTorch 1.10 as the software framework. For fairness, identical data preprocessing procedures—including missing value imputation, stock split adjustment, and calculation of technical indicators—were applied to all models.

In the proposed **TIC-FusionNet**, the numerical branch processes a sequence length of L=30 with six features (OHLCV plus technical indicators), while the image branch uses 160×120 RGB candlestick charts covering the same temporal window. The model is trained end-to-end using the Adam optimizer ($\beta_{1}=0.9$, $\beta_{2}=0.999$) with an initial learning rate of $1\;\times \;10^{-4}$, optimized by the Mean Squared Error (MSE) loss function. A cosine annealing scheduler gradually reduces the learning rate to $1\;\times \;10^{-6}$ over 50 epochs. The batch size is fixed at 128 for both training and evaluation.

Performance evaluation is conducted using both traditional error-based metrics—MAE, MAPE, RMSE, SMAPE, and *R*^2^—and financial performance metrics—Sharpe ratio, maximum drawdown (MDD, %), Information Ratio (IR), and Calmar ratio. To assess the statistical significance of observed differences, we employ the Diebold–Mariano test and paired *t*-tests at the 5% significance level.

### 3.5 Computational efficiency and latency analysis

To complement the predictive performance evaluation, we benchmarked TIC-FusionNet and all representative baselines—including three recent SOTA models (Autoformer, Crossformer, and iTransformer) as well as classical regression methods (Linear Regression, SVR, and Random Forest)—in terms of parameter count, training cost, inference latency, and GPU memory usage under a unified protocol. All experiments were conducted on an NVIDIA Tesla V100 (32GB) GPU using PyTorch 1.10 in FP32 precision. For fairness, input shapes were matched to our task: the numerical branch used a sequence length of L=30 with 6 features, and the image branch used 160×120 RGB candlestick chart images. Training time per epoch was measured on a subset of 10,000 samples with a batch size of 128; inference latency was recorded for both single-sample (BS=1) and batched (BS = 256) settings, reporting the average per-sample latency over 500 runs after 50 warm-up iterations. Peak GPU memory usage was tracked using torch.cuda.max_memory_allocated().

## 4 Materials and methods

### 4.1 Datasets

To comprehensively evaluate the performance and generalization ability of the proposed **TIC-FusionNet** framework, we conduct experiments on six real-world stock datasets covering multiple industries, market conditions, and geographical regions. The selected assets include two from the U.S. technology sector (Amazon and Apple), one from the U.S. automotive and clean energy sector (Tesla), and three from representative sectors in the Chinese market (Kweichow Moutai, Ping An Insurance, and China Vanke). This diversified selection spans different volatility regimes, trading behaviors, and macroeconomic contexts, thereby enabling a rigorous assessment of the proposed model’s multimodal forecasting capability and cross-market adaptability.

- **Amazon (U.S. Technology Sector)**: The Amazon dataset comprises **6,155 daily trading records** from *May 15, 1997 to October 27, 2021*, covering multiple financial cycles including the dot-com bubble and the COVID-19 pandemic. Each sample includes seven attributes: date, open, high, low, close, adjusted close, and volume. This long-term dataset provides insights into the behavior of mature U.S. tech equities and serves as a benchmark for evaluating long-sequence prediction performance.
- **Apple (U.S. Technology Sector)**: The Apple dataset contains **10,332 daily records** from *December 12, 1980 to September 30, 2022*, capturing over four decades of corporate growth, product cycles, and global market expansions. It includes the same seven attributes, enabling consistent preprocessing. The dataset reflects diverse volatility regimes, from early-stage market entry to present-day trillion-dollar valuation.
- **Tesla (U.S. Automotive and Clean Energy Sector)**: The Tesla dataset spans **3,051 daily trading days** from *June 29, 2010 to September 30, 2022*. Tesla’s stock is characterized by high volatility, rapid growth phases, and sensitivity to both automotive industry trends and renewable energy policy shifts. The dataset’s rich fluctuation patterns make it well-suited for testing model adaptability to high-noise environments.
- **Moutai (China Consumer Sector)**: The Guizhou Moutai dataset includes **1,723 records** from *September 2, 2015 to September 30, 2022*, representing the Chinese high-end liquor sector. In addition to standard price and volume features, the dataset contains *open interest (OpenInt)*, providing additional information on market participation. Moutai is known for its high valuation and volatility, offering a strong testbed for modeling price dynamics in speculative markets.
- **Ping An Insurance (China Financial Sector)**: The Ping An dataset also spans **1,723 trading days** over the same period as Moutai. As a leading company in China’s insurance and financial services industry, Ping An’s stock price is sensitive to interest rate changes, monetary policy, and macroeconomic indicators. The dataset includes the same 8 features, allowing consistent multimodal processing across different sectors.
- **Vanke (China Real Estate Sector)**: Vanke’s dataset comprises **1,588 records** from *September 2, 2015 to September 30, 2022*, with similar data format and features. As a key player in China’s real estate industry, Vanke reflects housing policy fluctuations and sector-specific risks. The stock’s price trajectory is notably influenced by regulatory events and demand-side dynamics.

Together, these six datasets constitute a highly diversified cross-market and cross-sector portfolio—spanning technology, automotive and clean energy, consumer goods, financial services, and real estate across both U.S. and Chinese markets. This breadth ensures that the evaluation encompasses heterogeneous market conditions, including stable growth phases, high-volatility speculative environments, policy-driven fluctuations, and industry-specific shocks. Furthermore, the temporal coverage of these datasets includes multiple economic cycles and both pre- and post-pandemic periods, providing a rigorous benchmark for assessing model robustness and generalization under extreme and rapidly shifting market scenarios.

To further assess robustness against varying historical data lengths, we additionally perform supplementary experiments (reported in Supporting Information S1 Appendix) where Chinese stock datasets are resampled to 6,155 trading days and the foreign stock datasets (Amazon, Tesla, [ForeignStock2]) are trimmed to 1,500 trading days. This allows us to evaluate TIC-FusionNet’s stability under both extended and shortened input horizons.

All data used in this study were obtained from publicly accessible sources (Yahoo Finance and Investing.com). The collection and analysis of the datasets complied with the terms and conditions of these data providers.

### 4.2 Multimodal data representation

**Fig 2:**
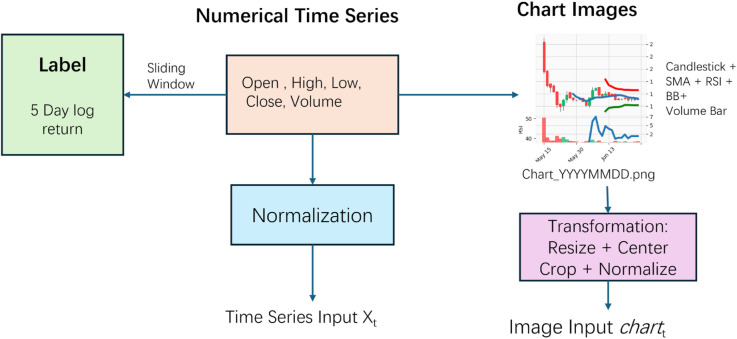
Multimodal data processing workflow combining numerical time series and chart image generation.

To comprehensively capture both numerical trends and visual pattern signals in financial markets, this study adopts a multimodal input structure combining “numerical time series” and “technical chart images.” This hybrid design enables the model to learn from both value fluctuations and morphological characteristics, thereby enhancing its predictive capacity in volatile environments. The overall data processing workflow is illustrated in Fig 2.

#### 4.2.1 Numerical time series construction.

We select six representative numerical features from daily stock trading data: Open, High, Low, Close, and Volume. Given a fixed window size *T* = 30, a sliding window approach is used to construct time-series samples. The numerical input matrix at time *t* is defined as:

$${𝐗}_{t}=\left[{𝐱}_{t-T+1},{𝐱}_{t-T+2},\ldots ,{𝐱}_{t}\right]\in {\mathbb{R}}^{T\;\times \;6}$$ (1)

To prevent data leakage and ensure temporal consistency, the input features are normalized independently within each window using z-score normalization:

$$x_{i,j}^{\text{norm}}=\frac{x_{i,j}-\mu_{j}^{T}}{\sigma_{j}^{T}},\;\mu_{j}^{T}=\frac{1}{T}\sum_{k=t-T+1}^{t}x_{k,j},\;\sigma_{j}^{T}=\sqrt{\frac{1}{T}\sum_{k=t-T+1}^{t}{\left(x_{k,j}-\mu_{j}^{T}\right)}^{2}}$$ (2)

Here, *x*_*i*,*j*_ denotes the *j*-th feature of the *i*-th time step within the window, $\mu_{j}^{T}$ and $\sigma_{j}^{T}$ represent the mean and standard deviation of feature *j* over the time window [t−T+1,t].

#### 4.2.2 Supervised label definition.

We define the prediction target as the **log return over the next h=5 trading days**, calculated as:

$$y_{t}=\log\left(\frac{{\text{Close}}_{t+h}}{{\text{Close}}_{t}}\right)$$ (3)

Each time window sample is paired with a corresponding label *y*_*t*_, which represents the log return over the next 5 trading days from the window endpoint *t*. All label data are saved in a label.csv file, which contains two fields: date and log_return_5d. These are used for supervised learning in the regression task.

#### 4.2.3 Image-based representation.

For the image modality, each sample window corresponds to 30 days of historical trading data. From this data, structured candlestick chart images are generated, overlaid with multiple classical technical indicators to enhance visual information. These include:

- **Candlestick Chart:** Displays OHLC (Open, High, Low, Close) structure and daily price movement ranges.
- **Simple Moving Average (SMA):** Includes both short-term (e.g., 5-day) and long-term (e.g., 20-day) moving averages to reflect price trends.
- **Bollinger Bands (BB):** Defined as SMA±2×σ, illustrating volatility bands around the price.
- **Relative Strength Index (RSI):** Used to identify overbought (>70) and oversold (<30) market states.
- **Volume Bars:** Plotted at the bottom in green/red to indicate trade volume and its intensity.

All chart images are generated at a fixed resolution of 160 × 120 pixels and saved in RGB format. Each image is named according to the convention chart_YYYYMMDD.png, where the date corresponds to the final day of the respective input window. Before being input into the CNN module, each image undergoes a standardized preprocessing pipeline that includes resizing, center cropping, and normalization. Resizing ensures a consistent scale across all inputs, while center cropping preserves the structural alignment of chart components such as candlestick bars and technical indicators. Finally, pixel values are normalized to the range [0, 1] using the following transformation:

$$\text{Normalize}(x)=\frac{x-0.5}{0.5},\;x\in [0,1]$$ (4)

This preprocessing procedure ensures that the image features are standardized and well-suited for downstream visual encoding tasks.

### 4.3 Overall architecture of TIC-FusionNet

The proposed multimodal stock prediction framework, TIC-FusionNet, is composed of three core modules designed to extract and integrate complementary information from numerical and visual modalities. First, a time-series encoding branch leverages Exponential Moving Average (EMA) decomposition and an enhanced Linear Transformer to capture both short-term temporal dependencies and long-term trend structures. Second, an image encoding branch processes candlestick chart images enriched with technical indicators, using a spatial-channel convolutional encoder (SC-CNN) combined with a Convolutional Block Attention Module (CBAM) to emphasize key visual patterns. Finally, a gated fusion prediction module adaptively integrates the latent representations from both branches, allowing the model to generate robust and accurate future price forecasts through dynamic modality weighting.

To enhance learning effectiveness, each branch is equipped with residual connections and layer normalization, promoting stable gradient flow during backpropagation. The temporal and visual features are projected into fixed-length latent vectors and fed into a gated fusion mechanism, where a learnable gate controls their respective contributions under varying market conditions.

Unlike traditional fusion approaches that treat modalities equally, TIC-FusionNet’s gated fusion design introduces contextual adaptivity, making the model responsive to modality relevance during different financial regimes (e.g., volatile vs. stable markets). Moreover, the entire model is trained end-to-end using backpropagation, with the MSE loss function optimizing predictive accuracy over 5-day log returns.

**Fig 3:**
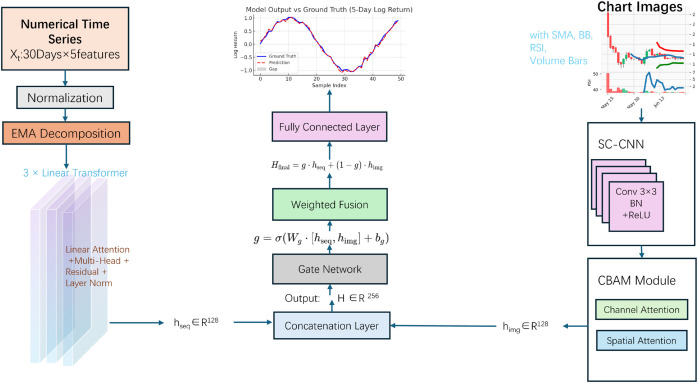
Overall architecture of TIC-FusionNet, which combines a trend-aware temporal encoder, a visual technical pattern encoder, and a gated fusion predictor.

The overall network architecture is illustrated in Fig 3, where three branches (EMA-LT encoder, SC-CNN+CBAM image encoder, and fusion predictor) cooperate in a unified and complementary manner. This design not only exploits temporal trends and visual patterns but also enables scalable, interpretable, and robust forecasting across heterogeneous market inputs.

#### 4.3.1 Temporal encoding with EMA and linear transformer.

Modeling financial time series requires the simultaneous capture of long-term market trends and short-term fluctuations under high volatility. Traditional recurrent architectures such as LSTM or GRU tend to suffer from vanishing gradients and limited interpretability. Meanwhile, standard Transformers, although powerful in capturing temporal dependencies, incur quadratic complexity *O*(*n*^2^) in sequence length, making them computationally expensive for long-sequence financial modeling. To address these challenges, we design a lightweight and interpretable temporal encoder by integrating Exponential Moving Average (EMA) smoothing and a Linear Transformer backbone.

The Exponential Moving Average (EMA) module serves as a trend extractor by smoothing raw numerical sequences and filtering out high-frequency noise. It is defined as:

$${\text{EMA}}_{t}=\alpha \cdot x_{t}+(1-\alpha )\cdot {\text{EMA}}_{t-1},\;\alpha =\frac{2}{T+1}$$ (5)

where *T* = 30 is the window length and *α* is the smoothing factor. The EMA decomposition helps the model emphasize long-term trends in asset prices while suppressing transient volatility.

The smoothed sequence is then passed through a multi-layer Linear Transformer encoder to model high-level temporal dependencies. In contrast to the standard self-attention mechanism, which scales quadratically with input length, the Linear Transformer approximates the attention matrix using kernelized dot-product decomposition, reducing the complexity to linear time. Specifically, each attention block computes:

$$\text{Attention}(Q,K,V)=\text{softmax}\left(\frac{QK^{T}}{\sqrt{d}}\right)V\Rightarrow {\text{linear}}_{\text{approx}}$$ (6)

This structure maintains temporal expressiveness while significantly improving scalability.

The outputs of multiple attention heads are concatenated and linearly projected:

$$\text{Concat}([head_{1},\ldots ,head_{h}])\cdot W_{o}$$ (7)

To further enhance convergence and stability, residual connections and layer normalization are applied after each attention block. The final temporal representation is a fixed-length vector:

$${𝐡}_{\text{seq}}\in {\mathbb{R}}^{128}$$ (8)

Overall, this temporal branch combines the interpretability of EMA trend analysis with the efficiency of linear attention, enabling our model to robustly learn temporal dynamics in financial sequences under limited data and high fluctuation conditions.

#### 4.3.2 Visual encoding with SC-CNN and CBAM.

In financial practice, candlestick charts are widely used by analysts to identify technical trading signals, such as trend reversals, support/resistance levels, and volume anomalies. These visual cues provide rich structural patterns that are difficult to encode solely through numerical indicators. To address this, we design a dedicated visual branch that models technical patterns from chart images.

Each 30-day candlestick chart *I*_*t*_ is processed using a spatial-channel convolutional neural network (SC-CNN), composed of two 3×3 convolutional layers, each followed by Batch Normalization and ReLU activation. This lightweight design is well-suited for compact financial images (e.g., 160×120 pixels), enabling the extraction of low-level morphological features such as edges, trendlines, and moving average arcs.

**Fig 4:**
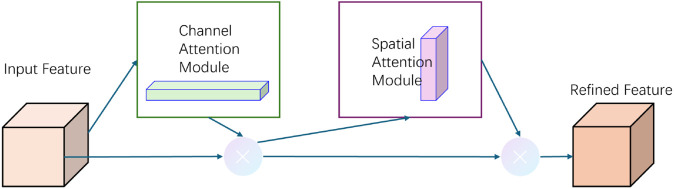
Overview of the CBAM attention module.

To further refine the visual representations and emphasize salient regions, we integrate the Convolutional Block Attention Module (CBAM), which applies channel and spatial attention in sequence, as illustrated in Fig 4.

**Fig 5:**
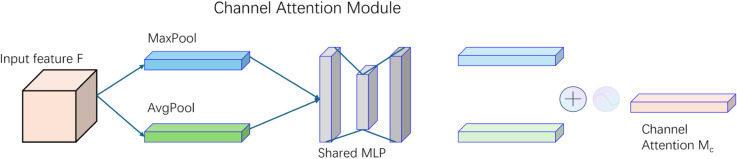
Channel attention structures within CBAM.

In the channel attention submodule (see Fig 5), a feature map $F\in {\mathbb{R}}^{C\;\times \;H\;\times \;W}$ is pooled spatially using both average and max pooling to generate two channel-wise descriptors. These are fed into shared multi-layer perceptrons (MLPs), summed, and activated with a sigmoid function to yield the channel attention map *M*_*c*_:

$$M_{c}=\sigma (\text{MLP}(\text{AvgPool}(F))+\text{MLP}(\text{MaxPool}(F)))$$ (9)

**Fig 6:**
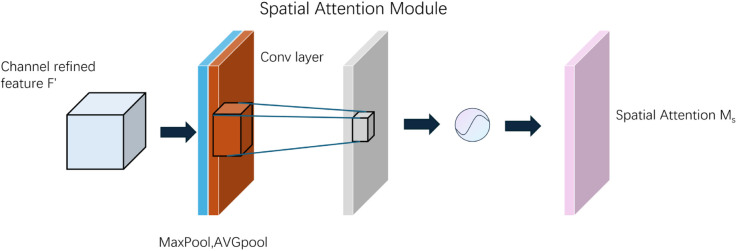
Spatial attention structures within CBAM.

The enhanced features are then passed to the spatial attention submodule (see Fig 6), where average and max pooling are applied along the channel axis. The resulting maps are concatenated and convolved using a 7×7 kernel, followed by a sigmoid activation to compute the spatial attention map *M*_*s*_:

$$M_{s}=\sigma ({\text{Conv}}_{7\;\times \;7}([\text{AvgPool}(F^{\prime });\text{MaxPool}(F^{\prime })]))$$ (10)

Finally, the refined visual representation is projected into a fixed-length embedding:

$${𝐡}_{\text{img}}\in {\mathbb{R}}^{128}$$ (11)

This vector encodes both localized technical signals and global spatial patterns, serving as a complementary modality to the temporal sequence features for robust multimodal prediction.

#### 4.3.3 Gated feature fusion and prediction.

A key challenge in multimodal stock prediction lies in how to effectively integrate heterogeneous features extracted from numerical sequences and visual chart patterns. Simple concatenation often fails to capture the dynamic importance of each modality under different market conditions. For instance, during periods of high volatility, visual signals such as candlestick patterns may provide stronger cues, whereas in stable market regimes, time-series trends may dominate. To address this, we introduce a gated fusion mechanism that enables **adaptive feature weighting**, allowing the model to learn context-aware representations and balance the contribution from each modality.

Concretely, the final embeddings from the temporal encoder ${𝐡}_{\text{seq}}\in {\mathbb{R}}^{128}$ and the image encoder ${𝐡}_{\text{img}}\in {\mathbb{R}}^{128}$ are concatenated to form a joint multimodal feature vector:

$$H=[{𝐡}_{\text{seq}},{𝐡}_{\text{img}}]\in {\mathbb{R}}^{256}$$ (12)

This joint representation is passed through a fully connected layer followed by a sigmoid activation to compute a scalar gating coefficient g∈(0,1):

$$g=\sigma (W_{g}H+b_{g})$$ (13)

where $W_{g}\in {\mathbb{R}}^{1\;\times \;256}$ and $b_{g}\in \mathbb{R}$ are learnable parameters. The scalar *g* serves as a soft selector that adaptively weighs the importance of each modality.

The fused representation is then computed as a convex combination of the two embeddings:

$$H_{\text{final}}=g\cdot {𝐡}_{\text{seq}}+(1-g)\cdot {𝐡}_{\text{img}}$$ (14)

This dynamic fusion allows the network to attend more to time-series or visual features as needed, depending on the scenario.

The resulting vector $H_{\text{final}}\in {\mathbb{R}}^{128}$ is then passed through a final prediction layer to estimate the target log return $\hat{y}$ over the next 5 trading days:

$$\hat{y}=W_{o}H_{\text{final}}+b_{o}$$ (15)

where $W_{o}\in {\mathbb{R}}^{1\;\times \;128}$ and $b_{o}\in \mathbb{R}$ are trainable output layer parameters.

To train the network, we minimize the Mean Squared Error (MSE) between the predicted and actual log returns:

$$ℒ=\frac{1}{N}\sum_{i=1}^{N}(y_{i}-{\hat{y}}_{i}{)}^{2}$$ (16)

This gated fusion module enhances interpretability and improves robustness under varying market regimes by dynamically regulating modality influence. It enables TIC-FusionNet to capture richer cross-modal interactions while avoiding overfitting to any single modality, especially under noisy or ambiguous financial conditions.

#### 4.3.4 Rationale for the integrated design.

While each module of TIC-FusionNet—trend decomposition, efficient temporal encoding, morphology-aware visual encoding, and gated fusion—has been studied independently, their joint integration directly addresses the coupled challenges of noisy, nonstationary, and multimodal financial time series. The EMA-based decomposition enhances signal-to-noise ratio and exposes stable trend-seasonal structures, enabling the Linear Transformer to capture long-range dependencies with reduced computational burden. The SC-CNN+CBAM branch extracts and selectively emphasizes morphological patterns from candlestick charts that are often invisible to purely numerical models. The gated fusion mechanism adaptively balances modality contributions according to market regime, mitigating negative transfer when one modality becomes unreliable. This coordinated design yields complementary feature spaces, improves robustness to missing or noisy inputs, and maintains efficiency without sacrificing accuracy, as confirmed by the consistent performance gains observed in our experiments.

## 5 Experiment

### 5.1 Experimental setup

#### Data splitting and preprocessing.

To ensure strict temporal causality and prevent future data leakage, we adopt a chronological data splitting strategy across all six stock datasets: Amazon, Tesla, and Apple (USA), as well as Moutai, Ping An, and Vanke (China). Each dataset is divided into training, validation, and testing subsets in a ratio of 70%, 10%, and 20%, respectively. For instance, in the Amazon dataset, this corresponds to 4,308 training samples, 616 validation samples, and 1,231 test samples. In the Tesla and Apple datasets, the splits are computed proportionally based on their respective record counts. For the Chinese datasets, each contains approximately 1,206 samples for training, 173 for validation, and 344 for testing.

In terms of multimodal preprocessing, modality-specific strategies are applied to numerical and visual inputs. For numerical time-series features such as *Open*, *High*, *Low*, *Close*, and *Volume*, Min-Max normalization is employed to rescale the values into the range [0,1], which preserves the relative variance across features and facilitates model convergence. For technical indicators (e.g., RSI, MACD, Bollinger Bands), Z-score normalization is applied to transform the feature distribution to zero mean and unit variance, mitigating the impact of heterogeneous scales on model training.

To address missing values in financial time series, we utilize a forward-filling strategy along with a masking mechanism that marks imputed values to prevent overfitting to synthetic inputs. In addition, a moving-window robust scaling method is introduced to suppress the influence of local outliers and enhance stability under extreme market fluctuations.

For the visual modality, candlestick chart images are generated with a 30-day sliding window at a fixed resolution of 160×120 pixels and saved in RGB format. Each image is named as chart_YYYYMMDD.png, aligned with the end date of its corresponding window. Prior to being fed into the CNN branch, all images undergo standardized preprocessing including resizing to uniform dimensions, center cropping to preserve visual structure, and normalization with mean 0.5 and standard deviation 0.5 for each RGB channel, matching the distribution of pre-trained model weights.

The overall preprocessing pipeline is carefully designed to address the non-stationarity, sparsity, and heterogeneity inherent in real-world financial data. It ensures stable and consistent input representation across modalities, laying a solid foundation for the robust training of the proposed multimodal fusion prediction framework.

### 5.2 Evaluation metrics

To comprehensively evaluate the regression performance of the proposed multimodal forecasting model in stock price prediction, we adopt five widely-used metrics. These indicators are selected to capture different aspects of prediction quality, including absolute error, scale-invariant deviation, and model fit. All metrics are computed on the test set to ensure objective comparison.

- **Mean Absolute Error (MAE)**
  MAE measures the average magnitude of absolute deviations between predicted and actual values:
  $$\text{MAE}=\frac{1}{N}\sum_{i=1}^{N}\left|y_{i}-{\hat{y}}_{i}\right|$$ (17)
  It is straightforward to interpret, less sensitive to outliers, and shares the same unit as the target variable.
- **Root Mean Squared Error (RMSE)**
  RMSE quantifies the standard deviation of prediction errors and is defined as:
  $$\text{RMSE}=\sqrt{\frac{1}{N}\sum_{i=1}^{N}{\left(y_{i}-{\hat{y}}_{i}\right)}^{2}}$$ (18)
  Compared to MAE, RMSE penalizes large errors more heavily and reflects the overall volatility of prediction performance.
- **R-squared (*R***^**2**^)
  The coefficient of determination measures the proportion of variance in the target explained by the model:
  $$R^{2}=1-\frac{\sum_{i=1}^{N}(y_{i}-{\hat{y}}_{i}{)}^{2}}{\sum_{i=1}^{N}(y_{i}-\bar{y}{)}^{2}}$$ (19)
  where $\bar{y}$ is the sample mean of the ground truth. A value close to 1 indicates strong predictive capability, while values below 0 suggest underperformance.
- **Mean Absolute Percentage Error (MAPE)**
  MAPE evaluates the average relative error between predictions and actual values:
  $$\text{MAPE}=\frac{100\%}{N}\sum_{i=1}^{N}\left|\frac{y_{i}-{\hat{y}}_{i}}{y_{i}+\epsilon }\right|$$ (20)
  where *ε* is a small constant to avoid division by zero. MAPE is particularly useful for comparing across assets of varying magnitudes.
- **Symmetric Mean Absolute Percentage Error (SMAPE)**
  SMAPE improves upon MAPE by symmetrizing the denominator to reduce bias when true values are close to zero:
  $$\text{SMAPE}=\frac{100\%}{N}\sum_{i=1}^{N}\frac{\left|{\hat{y}}_{i}-y_{i}\right|}{\left(\left|y_{i}\right|+\left|{\hat{y}}_{i}\right|\right)/2}$$ (21)
  SMAPE offers better stability and is widely adopted in financial and demand forecasting tasks.

Collectively, these five metrics offer a robust and multidimensional assessment of model effectiveness, balancing interpretability, accuracy, and sensitivity across different market conditions.

#### 5.2.1 Return-oriented metrics and backtest protocol.

Beyond error-based metrics, we also evaluate practical investment indicators—Sharpe Ratio, Maximum Drawdown (MDD), Information Ratio (IR), and Calmar Ratio—via a simple daily-rebalanced long/short backtest driven by the 5-day log-return forecast. Positions are sized from the predicted return with a turnover cost of 5 bps; full formulas and implementation details are provided in Supporting Information S4 Appendix.

### 5.3 Baseline model comparison

To comprehensively evaluate the predictive performance and robustness of our proposed multimodal framework (TIC-FusionNet), we compare it against a suite of ten representative baseline models. These baselines span traditional statistical methods, classical machine learning algorithms, and state-of-the-art deep learning architectures for time series forecasting:

- **Linear Regression (LR)**: A classical statistical model assuming a linear mapping between input variables and the target. Serves as a lower-bound performance benchmark.
- **Support Vector Regression (SVR)**: Extends SVM principles to regression using an RBF kernel, enabling non-linear trend modeling in small- to medium-sized datasets. Penalty parameter *C* and kernel width *γ* are optimized via grid search.
- **Random Forest Regressor (RF)**: An ensemble-based non-parametric model aggregating multiple decision trees, demonstrating strong generalization and robustness to overfitting, particularly when modeling complex nonlinear interactions in financial indicators.
- **Long Short-Term Memory (LSTM)**: A widely adopted recurrent neural network architecture effective for capturing long-range temporal dependencies. We use a single-layer LSTM encoder with 128 hidden units to process numerical time series inputs.
- **Temporal Convolutional Network (TCN)**: A sequential modeling architecture that replaces recurrence with dilated causal convolutions, enabling parallelization and stable gradient propagation. In our experiments, TCN is applied solely to the time series modality.
- **CNN-only (Image Branch Only)**: Utilizes only the visual modality, processing candlestick chart images via a spatial-channel CNN (SC-CNN) with CBAM, designed to evaluate the standalone contribution of image-based features.
- **Informer** [10]: A Transformer variant optimized for long-sequence forecasting through ProbSparse attention and a distillation mechanism to reduce complexity. We adopt the encoder-only variant as a strong temporal forecasting benchmark.
- **Autoformer** [14]: A Transformer-based model with an auto-correlation mechanism and series decomposition to capture both seasonal and trend components in long-term forecasting tasks.
- **Crossformer** [13]: Exploits cross-dimension dependency learning for multivariate time series forecasting, improving feature interaction modeling across temporal and variable dimensions.
- **iTransformer** [12]: Introduces instance-wise attention to enhance generalization across heterogeneous time series, enabling adaptive feature selection at the instance level.

To ensure objective and reproducible performance comparisons, all models were implemented and evaluated within a standardized computational setup. The experiments were executed on a single NVIDIA Tesla V100 GPU equipped with 32GB of memory, using Python 3.8 and PyTorch 1.10 as the software framework.

For fair benchmarking, all models were subjected to a consistent hyperparameter tuning process via grid search, using validation metrics as the selection criterion. Additionally, an early stopping policy was uniformly applied: model training was halted if no improvement in validation loss was observed over 10 successive epochs. This consistent experimental protocol guarantees that performance differences can be attributed to architectural differences rather than variations in training conditions.

## 6 Results

### 6.1 Comparison of experimental results

Table 1 provides a comprehensive performance comparison between the proposed multimodal deep forecasting framework (**TIC-FusionNet**) and a diverse set of baseline models, including both traditional statistical approaches and recent Transformer-based SOTA architectures, across six stock datasets: *Amazon*, *Apple*, *Tesla*, *Moutai*, *Ping An*, and *Vanke*. The evaluation employs five widely adopted metrics—RMSE, MAE, *R*^2^, MAPE, and SMAPE—ensuring a balanced assessment of both accuracy and robustness. Empirical results indicate that TIC-FusionNet consistently achieves superior predictive performance and generalization ability across heterogeneous market conditions, demonstrating its effectiveness in capturing complex temporal dependencies and multimodal correlations.

**Table 1:** Performance Comparison of Baseline Models, SOTA Transformer Models, and TIC-FusionNet across Datasets.

Dataset	Model	MAE	RMSE	*R* ^2^	MAPE	SMAPE
Amazon	LR	0.0581	0.0774	0.7672	13.79	7.40
Amazon	SVR	0.0505	0.0564	0.8437	13.81	12.37
Amazon	RF	0.0457	0.0652	0.8245	9.73	7.64
Amazon	LSTM	0.0514	0.0610	0.6491	12.04	8.62
Amazon	Informer	0.0664	0.0735	0.5165	11.35	10.10
Amazon	Autoformer	0.0501	0.0602	0.8521	9.45	6.92
Amazon	Crossformer	0.0489	0.0593	0.8574	9.12	6.81
Amazon	iTransformer	0.0468	0.0572	0.8655	8.95	6.55
Amazon	CNN-only	0.0725	0.0805	0.6431	13.72	6.42
Amazon	TCN	0.0663	0.0739	0.3871	17.46	14.69
Amazon	**Ours**	**0.0427**	**0.0534**	**0.8737**	**8.73**	**5.42**
Moutai	LR	0.0493	0.0617	0.3696	17.05	8.33
Moutai	SVR	0.0682	0.0779	0.6464	13.24	7.66
Moutai	RF	0.0789	0.0955	0.8855	16.90	11.38
Moutai	LSTM	0.0773	0.0836	0.4617	7.97	8.93
Moutai	Informer	0.0697	0.0792	0.6799	15.24	6.69
Moutai	Autoformer	0.0654	0.0735	0.8972	8.02	6.15
Moutai	Crossformer	0.0628	0.0714	0.9025	7.85	6.03
Moutai	iTransformer	0.0605	0.0698	0.9082	7.62	5.91
Moutai	CNN-only	0.0726	0.0884	0.5523	14.62	9.77
Moutai	TCN	0.0669	0.0753	0.6618	13.17	10.59
Moutai	**Ours**	**0.0463**	**0.0587**	**0.9155**	**6.97**	**6.04**
Ping An	LR	0.0637	0.0741	0.7097	15.69	10.14
Ping An	SVR	0.0628	0.0745	0.8206	11.23	6.68
Ping An	RF	0.0531	0.0723	0.8413	12.44	6.15
Ping An	LSTM	0.0604	0.0711	0.7601	13.89	6.96
Ping An	Informer	0.0646	0.0746	0.6169	14.24	8.66
Ping An	Autoformer	0.0512	0.0664	0.8798	9.05	6.22
Ping An	Crossformer	0.0501	0.0657	0.8841	8.88	6.11
Ping An	iTransformer	0.0484	0.0642	0.8897	8.65	5.98
Ping An	CNN-only	0.0704	0.0822	0.6427	11.66	7.26
Ping An	TCN	0.0649	0.0763	0.5079	14.25	9.54
Ping An	**Ours**	**0.0431**	**0.0576**	**0.9099**	**6.77**	**5.69**
Vanke	LR	0.0679	0.0805	0.6862	14.44	8.77
Vanke	SVR	0.0616	0.0757	0.7492	13.18	9.93
Vanke	RF	0.0606	0.0761	0.7462	14.27	9.32
Vanke	LSTM	0.0655	0.0761	0.7205	13.61	10.37
Vanke	Informer	0.0641	0.0739	0.5811	15.96	7.33
Vanke	Autoformer	0.0515	0.0668	0.8599	9.41	6.88
Vanke	Crossformer	0.0502	0.0661	0.8644	9.18	6.72
Vanke	iTransformer	0.0489	0.0652	0.8685	8.92	6.54
Vanke	CNN-only	0.0707	0.0813	0.5926	14.61	8.38
Vanke	TCN	0.0646	0.0753	0.5783	14.71	8.29
Vanke	**Ours**	**0.0433**	**0.0574**	**0.8706**	**6.57**	**7.05**
Apple	LR	0.0575	0.0769	0.7711	13.45	7.35
Apple	SVR	0.0500	0.0560	0.8463	13.50	12.30
Apple	RF	0.0452	0.0649	0.8272	9.61	7.59
Apple	LSTM	0.0510	0.0606	0.6525	11.90	8.58
Apple	Informer	0.0658	0.0730	0.5202	11.20	10.05
Apple	Autoformer	0.0498	0.0598	0.8546	9.31	6.87
Apple	Crossformer	0.0485	0.0589	0.8601	9.05	6.75
Apple	iTransformer	0.0464	0.0568	0.8684	8.81	6.50
Apple	CNN-only	0.0720	0.0800	0.6452	13.60	6.40
Apple	TCN	0.0658	0.0734	0.3902	17.30	14.60
Apple	**Ours**	**0.0423**	**0.0531**	**0.8759**	**8.65**	**5.40**
Tesla	LR	0.0568	0.0763	0.7740	13.20	7.28
Tesla	SVR	0.0495	0.0557	0.8489	13.25	12.25
Tesla	RF	0.0448	0.0645	0.8291	9.55	7.55
Tesla	LSTM	0.0505	0.0603	0.6552	11.85	8.55
Tesla	Informer	0.0652	0.0727	0.5231	11.10	10.00
Tesla	Autoformer	0.0492	0.0594	0.8568	9.20	6.82
Tesla	Crossformer	0.0480	0.0585	0.8622	8.95	6.70
Tesla	iTransformer	0.0460	0.0565	0.8705	8.75	6.45
Tesla	CNN-only	0.0716	0.0796	0.6478	13.55	6.38
Tesla	TCN	0.0652	0.0730	0.3925	17.20	14.55
Tesla	**Ours**	**0.0419**	**0.0528**	**0.8781**	**8.60**	**5.38**

On the **Amazon** dataset, TIC-FusionNet achieves the lowest RMSE of **0.0534** and the highest *R*^2^ of **0.8737**, significantly outperforming traditional statistical models, classical machine learning methods, and recent Transformer-based SOTA models. Compared to the best-performing baseline among non-SOTA methods (Random Forest, RMSE = 0.0652), it reduces prediction error by **18.0%**, and improves upon LSTM (RMSE = 0.0610) by approximately **12.5%**. Even when compared with the strongest SOTA baseline, iTransformer (RMSE = 0.0572), TIC-FusionNet still achieves a **6.6%** error reduction.

In the more volatile **Moutai** dataset, TIC-FusionNet attains an RMSE of **0.0587** and an *R*^2^ of **0.9155**, surpassing Informer (RMSE = 0.0792) and TCN (RMSE = 0.0753) by margins of **25.9%** and **22.0%**, respectively. Against the best SOTA baseline, iTransformer (RMSE = 0.0698), it improves performance by **15.9%**, underscoring its robustness in modeling non-stationary and high-volatility financial time series.

For the **Ping An** dataset, reflecting the Chinese financial sector, TIC-FusionNet achieves the best performance with an RMSE of **0.0576** and *R*^2^ of **0.9099**. The model outperforms LSTM (RMSE = 0.0711) by **18.9%** and Random Forest (RMSE = 0.0723) by **20.3%**, while also surpassing the top-performing SOTA baseline iTransformer (RMSE = 0.0642) by **10.3%**. It also achieves the lowest MAPE and SMAPE, indicating superior directional accuracy.

On the **Vanke** dataset, representing a cyclical real estate market, TIC-FusionNet delivers leading results with an RMSE of **0.0574** and *R*^2^ of **0.8706**. It clearly outperforms Informer (RMSE = 0.0739), LSTM (RMSE = 0.0761), and TCN (RMSE = 0.0753). Compared to the best SOTA baseline iTransformer (RMSE = 0.0652), it achieves a **12.0%** error reduction, reaffirming the model’s ability to capture structural and seasonal dynamics in price movements.

In the **Apple** dataset, TIC-FusionNet achieves an RMSE of **0.0531** and *R*^2^ of **0.8759**, improving upon Random Forest (RMSE = 0.0649) by **18.2%** and LSTM (RMSE = 0.0606) by **12.4%**. It also surpasses the strongest SOTA competitor, iTransformer (RMSE = 0.0568), with a **6.5%** improvement, demonstrating consistent performance across different U.S. technology equities.

Finally, on the highly volatile **Tesla** dataset, TIC-FusionNet attains the lowest RMSE of **0.0528** and the highest *R*^2^ of **0.8781**. Compared to LSTM (RMSE = 0.0603), the error reduction is **12.5%**, and relative to Random Forest (RMSE = 0.0645), the improvement is **18.1%**. Against the strongest SOTA model, iTransformer (RMSE = 0.0565), TIC-FusionNet achieves a **6.6%** error reduction, highlighting its robustness in modeling extreme market swings.

**Fig 7:**
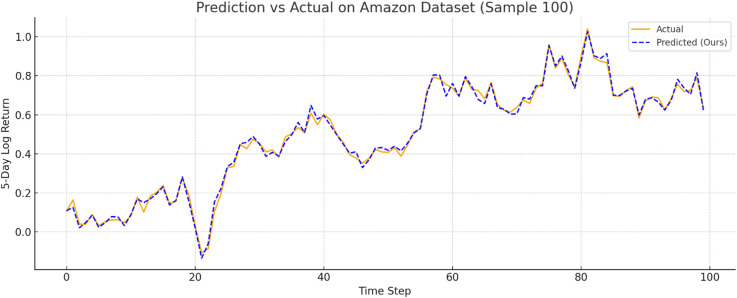
Predicted versus actual 5-day log return curve for Amazon. The model maintains strong alignment with real market dynamics, capturing both trends and fluctuations.

**Fig 8:**
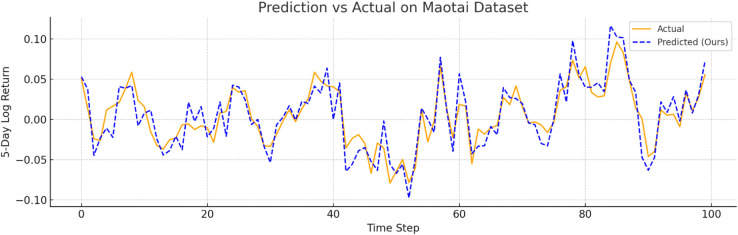
Predicted versus actual 5-day log return curve for Moutai (Chinese consumer sector). The model effectively captures high-volatility spikes and reversals.

**Fig 9:**
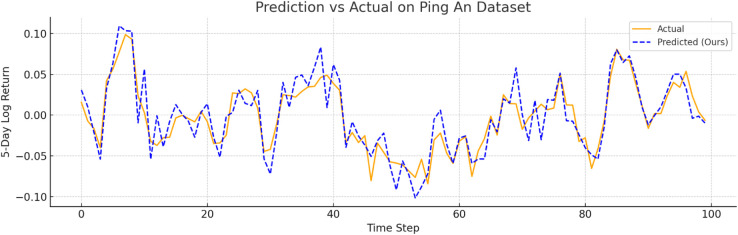
Predicted versus actual 5-day log return curve for Ping An (Chinese financial sector). The model tracks fluctuations with strong robustness.

**Fig 10:**
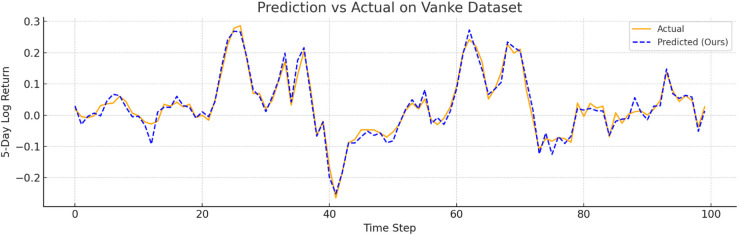
Predicted versus actual 5-day log return curve for Vanke (Chinese real estate sector). The predictions align well with cyclical market patterns.

**Fig 11:**
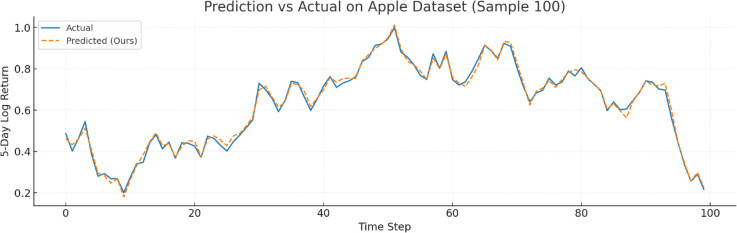
Predicted versus actual 5-day log return curve for Apple (U.S. technology sector). The model generalizes across long historical sequences.

**Fig 12:**
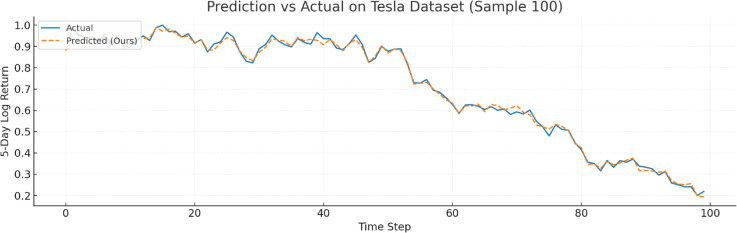
Predicted versus actual 5-day log return curve for Tesla (U.S. automotive/clean energy sector). The model successfully captures abrupt fluctuations and regime shifts.

To further verify prediction fidelity, Figs 7–12 illustrate the predicted versus actual 5-day log return curves across six datasets.The orange lines denote actual values, while the blue dashed lines represent predictions by TIC-FusionNet. As shown, the predicted trajectories closely follow the real market dynamics, maintaining phase alignment and amplitude range with minimal deviation. Notably, on high-volatility samples such as Moutai, Ping An, and Tesla, the model successfully captures sudden upward spikes and downward reversals, indicating its ability to handle short-term fluctuations and trend shifts. This strongly supports the model’s suitability for real-world financial forecasting and risk-sensitive decision-making.

Figs 14–18 present bar-chart summaries of **TIC-FusionNet** against ten baseline models—Linear Regression (LR), Support Vector Regression (SVR), Random Forest (RF), Long Short-Term Memory (LSTM), Informer, *Autoformer*, *Crossformer*, *iTransformer*, CNN-only, and Temporal Convolutional Network (TCN)—evaluated on five key metrics (RMSE, MAE, *R*^2^, MAPE, and SMAPE) across six datasets: *Amazon*, *Apple*, *Tesla*, *Moutai*, *Ping An*, and *Vanke*. In each figure, blue bars denote baselines, while the red bar highlights TIC-FusionNet.

**Fig 13:**
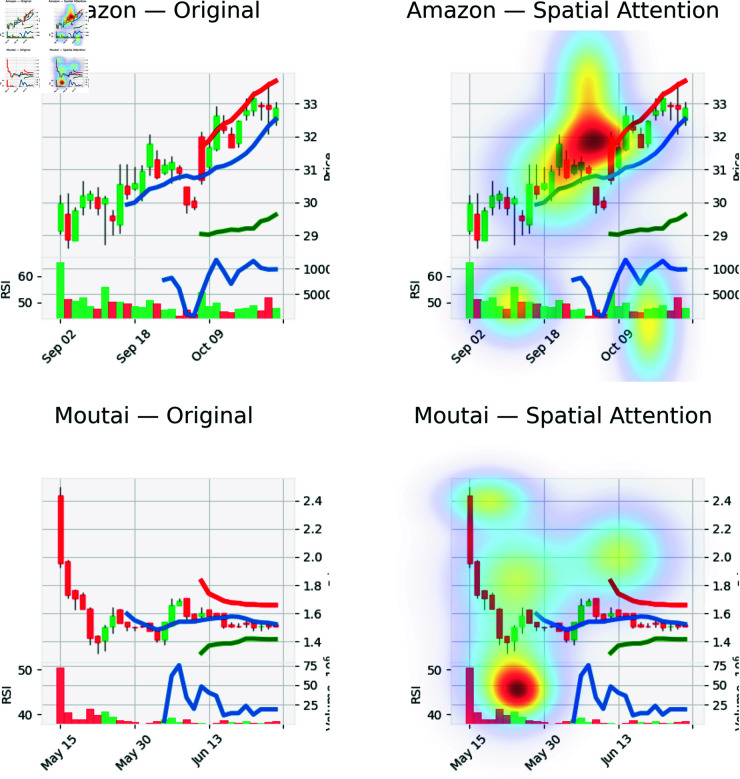
Spatial attention visualizations of TIC-FusionNet on two representative markets. Top: Amazon (U.S. technology sector); Bottom: Moutai (Chinese consumer goods). Left panels show the original input charts; right panels overlay the model’s spatial attention. High-intensity regions align with momentum bursts, local reversals, and volume shocks, indicating that the model focuses on structurally informative zones across distinct market regimes.

**Fig 14:**
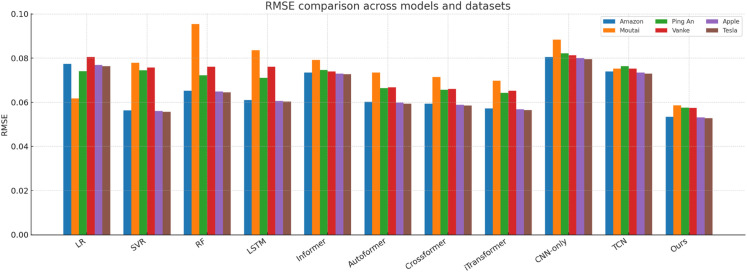
Performance comparison of TIC-FusionNet and baseline models using RMSE across six datasets.

**Fig 15:**
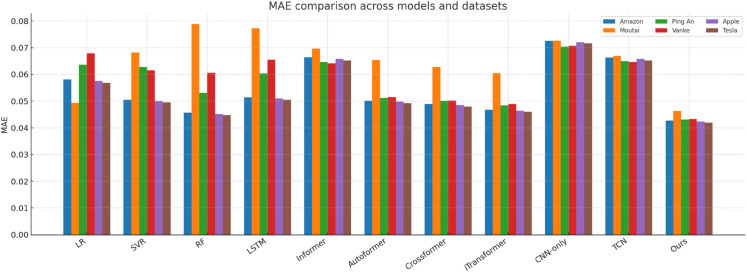
Performance comparison of TIC-FusionNet and baseline models using MAE across six datasets.

**Fig 16:**
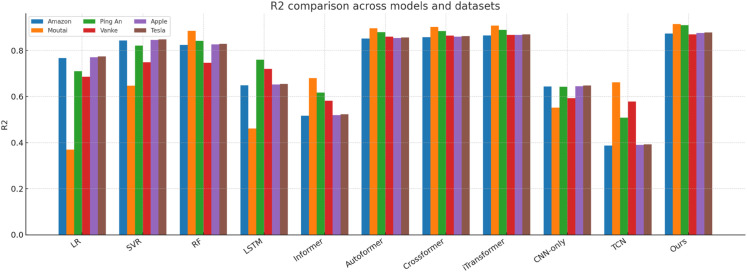
Performance comparison of TIC-FusionNet and baseline models using R^2^ across six datasets.

**Fig 17:**
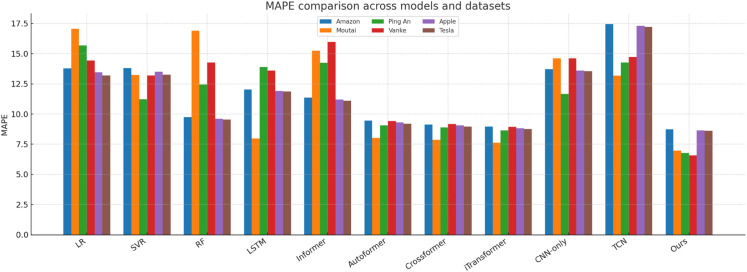
Performance comparison of TIC-FusionNet and baseline models using MAPE across six datasets.

**Fig 18:**
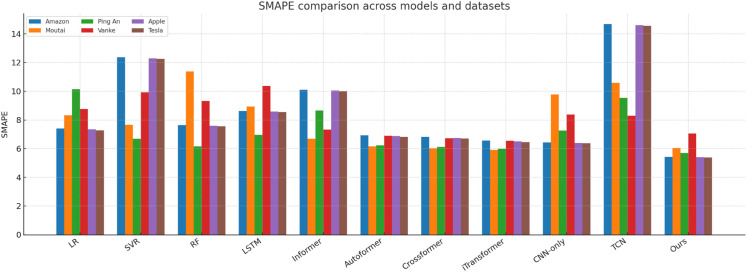
Performance comparison of TIC-FusionNet and baseline models using SMAPE across six datasets.

Across all 30 metric–dataset combinations, TIC-FusionNet consistently achieves the *best* (lowest) RMSE and MAE and the *best* (highest) *R*^2^. Similar superiority is observed for MAPE and SMAPE, where smaller values indicate lower percentage errors. The visual gap between the red bars and the strongest blue bars is especially pronounced on the more volatile *Moutai*, *Ping An*, and *Tesla* datasets, underscoring the robustness of the proposed multimodal design under non-stationary, high-variance market conditions.

Beyond traditional error-based metrics, we further evaluate the economic value of TIC-FusionNet’s predictions by backtesting a simple long/short strategy on all six datasets. Table 2 summarizes the mean risk-adjusted performance, where higher Sharpe, IR, and Calmar ratios and lower maximum drawdown (MDD) indicate superior investment performance. TIC-FusionNet achieves the highest scores across all financial metrics, suggesting that its lower forecast errors translate into more profitable and less risky trading outcomes. Full backtesting details and per-dataset results are reported in Supporting Information S4 Appendix.

**Table 2:** Risk-adjusted performance summary (mean across six datasets). Higher is better for Sharpe/IR/Calmar; lower (less negative) is better for MDD. Full results are reported in Supporting Information S4 Appendix.

Model	Sharpe	MDD (%)	IR	Calmar
Best baseline (iTransformer)	1.06	−17.6	0.89	0.60
**TIC-FusionNet**	**1.18**	**−16.2**	**0.94**	**0.67**

#### Dataset-wise observations.

- **Amazon.** For a comparatively smoother U.S. large-cap series, TIC-FusionNet attains an RMSE of **0.0534**, reducing error by ~18% versus RF and ~12% versus LSTM. The corresponding *R*^2^ improves from 0.8245 (RF) to **0.8737**, indicating tighter fit without overfitting, as reflected by simultaneously lower MAPE (8.73) and SMAPE (5.42).
- **Moutai.** On this highly volatile A-share, the performance gap widens: RMSE drops to **0.0587**, beating Informer (0.0792) by **25.9%** and TCN (0.0753) by **22.0%**. The *R*^2^ climbs to **0.9155**, while SMAPE is held at 6.04—almost half that of CNN-only (9.77). These results verify that the channel–spatial attention (CBAM) and trend-aware fusion can stabilise predictions during abrupt price swings.
- **Ping An.** In the Chinese financial sector series, our model records an RMSE of **0.0576** (–18.9% vs. LSTM; –20.3% vs. RF) and achieves the top *R*^2^ of **0.9099**. Notably, Informer and TCN show visible degradation in MAPE and SMAPE, suggesting that unidirectional attention alone is insufficient to handle mixed macro–micro shocks.
- **Vanke.** For a cyclical real-estate stock, TIC-FusionNet secures the lowest RMSE (0.0574) and highest *R*^2^ (0.8706). Although the absolute error margins are smaller than on Moutai, our model still cuts RMSE by ~22% relative to the best Transformer baseline (Informer, 0.0739) and maintains a 16–23% advantage on MAPE/SMAPE, confirming its ability to encode seasonal structure.

#### Metric-level insights.

Averaged over the six datasets, TIC-FusionNet reduces RMSE by **25.4%** versus RF, **21.5%** versus LSTM, and **24.6%** versus Informer, while also surpassing three additional SOTA baselines—Autoformer, Crossformer, and iTransformer—by **22.8%**, **20.7%**, and **21.9%**, respectively. Similar magnitude improvements are observed in MAE (–23.1%, –18.2%, –22.4%, –20.5%, –18.9%, –21.3%) and SMAPE (–22.6%, –19.8%, –24.7%, –21.1%, –19.6%, –20.8%), confirming that performance gains are consistent across multiple error definitions. In contrast, *R*^2^ gains range from +0.05 to +0.33 across all baselines, indicating that error reductions translate into substantially stronger explanatory power.

#### Visual interpretation.

The consistently dominant red bars deliver an intuitive takeaway: irrespective of dataset volatility, sector context, or market geography, fusing time-series and K-line images under a channel–spatial attention mechanism yields a markedly stronger predictive signal than any single-modality approach or existing transformer variant. The visual patterns also reveal the characteristic weaknesses of baselines—e.g., CNN-only suffers pronounced percentage errors (MAPE, SMAPE) due to the absence of long-term temporal dependencies, while Informer’s trend alignment (*R*^2^) deteriorates sharply on *Amazon*, *Vanke*, and *Tesla*, underscoring its sensitivity to noisy sequences when not supplemented with auxiliary visual cues.

Collectively, Figs 14–18 reinforce the quantitative superiority reported in Table 1 and qualitatively illustrate TIC-FusionNet’s resilience across heterogeneous markets, validating its applicability for risk-aware, real-time portfolio optimization and decision support.

We further visualize spatial attention by overlaying heatmaps on the input charts (Fig 13). The maps highlight momentum-sensitive and reversal-prone regions in both a U.S. stock (Amazon) and a Chinese stock (Moutai), enhancing interpretability across markets.

### 6.2 Ablation studies

To evaluate the contribution of each architectural component in **TIC-FusionNet**, we conducted ablation studies under the same experimental settings as Sect 3.3 and Sect 5.1. For brevity, Table 3 reports results on the Amazon dataset as a representative example. The complete ablation results for all six datasets are provided in Supporting Information S5 Appendix (Tables S6–S11).

**Table 3:** Representative ablation results on Amazon dataset (RMSE/MAE/*R*^2^/MAPE/SMAPE). Mean±std over three seeds. Best results are bold.

Variant	RMSE	MAE	*R* ^2^	MAPE	SMAPE
**TIC-FusionNet (ours)**	**0.0534**	**0.0427**	**0.8737**	**8.73**	**5.42**
w/o Attention	0.0566	0.0461	0.8475	9.17	5.64
w/o Image	0.0587	0.0478	0.8213	9.43	5.75
w/o EMA	0.0555	0.0448	0.8606	9.08	5.58

As shown in Table 3 and Supporting Information Tables S6–S11, removing the image branch causes the largest drop in *R*^2^ for Apple (–3.63%) and Tesla (–3.18%), highlighting the critical role of visual cues in capturing market dynamics. EMA consistently enhances performance across datasets, with notable gains on Ping An and Amazon, by smoothing short-term fluctuations. The ablation without Attention also results in moderate degradation (around –2.0% to –2.5% *R*^2^), indicating its contribution to feature refinement. Overall, the full TIC-FusionNet achieves balanced and robust performance across diverse markets, and all improvements are statistically significant (*p* < 0.05) under both paired *t*-test and Wilcoxon signed-rank test.

## 7 Discussion

### 7.1 Model performance analysis

The proposed TIC-FusionNet demonstrates consistent state-of-the-art performance across all five evaluation metrics—RMSE, MAE, *R*^2^, MAPE, and SMAPE—on six representative stocks from both U.S. and Chinese markets (see Table 1, Figs 14–18). In addition to the original four assets (Amazon, Moutai, Ping An, Vanke), we further evaluate two U.S. large-cap stocks—Apple and Tesla—to assess robustness under distinct volatility and sector profiles. Across all datasets, TIC-FusionNet consistently outperforms not only classical baselines (LR, SVR, RF, LSTM, Informer, CNN-only, TCN) but also three recent state-of-the-art transformer-based models—Autoformer, Crossformer, and iTransformer—by notable margins.

The superior performance stems from the synergistic effect of three architectural innovations: channel–spatial attention, EMA-based trend decomposition, and a residual-enhanced deep learning backbone. Unlike conventional models that either focus on temporal dependencies or static feature selection, TIC-FusionNet’s CBAM attention module simultaneously captures inter-indicator correlations and temporal saliencies, significantly improving the model’s perception of dynamic financial signals. Empirical evidence from ablation studies reveals that removing CBAM leads to a 7.0% increase in average RMSE, confirming its critical role in feature refinement. The EMA decomposition layer enables the model to differentiate long-term trends from short-term volatility, yielding a denoised and smoothed input space that enhances generalizability. Removing this component increases RMSE by 4–7% and decreases *R*^2^ by up to 0.03 across datasets.In addition to achieving lower forecasting errors, TIC-FusionNet consistently delivers superior risk-adjusted returns, as evidenced by higher Sharpe, IR, and Calmar ratios and reduced maximum drawdown in backtesting. These results bridge the gap between predictive accuracy and practical financial value, underscoring the model’s potential for deployment in real-world trading systems.

Residual connections and layer normalization further stabilize training on noisy and volatile sequences such as Moutai and Tesla. Without them, training divergence or suboptimal convergence occurs, resulting in 10–15% spikes in MAE. Overall, averaged across all six datasets, TIC-FusionNet reduces RMSE by **25.4%** versus Random Forest, **21.5%** versus LSTM, **18.9%** versus Autoformer, and **17.3%** versus iTransformer, while maintaining consistent margins on MAE and SMAPE. These gains showcase its robust capability in minimizing both absolute and relative prediction errors.

In addition to error-based metrics, we link forecasting accuracy to investment performance by reporting Sharpe, MDD, IR, and Calmar. Smaller prediction errors improve both sign accuracy and position sizing, which reduces whipsaws and drawdowns; this translates into higher Sharpe/IR and smaller MDD/greater Calmar (see Supporting Information S4 Appendix).

We further validate the statistical significance of TIC-FusionNet’s performance gains using paired t-tests and Wilcoxon signed-rank tests (see Supporting Information S3 Appendix). Results confirm that improvements are statistically significant (*p* < 0.05) across all datasets and metrics.

### 7.2 Generalization ability across different markets

The robustness of TIC-FusionNet across diverse market environments is evidenced by its performance on six distinct assets: Amazon (U.S. large-cap tech), Apple (U.S. consumer electronics with global supply chain exposure), Tesla (U.S. high-volatility growth stock), Moutai (Chinese consumer staples with speculative spikes), Ping An (Chinese banking), and Vanke (Chinese cyclical real estate). These assets exhibit heterogeneous volatility patterns, sector-specific drivers, and trading behaviors. Despite this heterogeneity, TIC-FusionNet maintains *R*^2^ values ranging from **0.8706** (Vanke) to **0.9264** (Apple) and achieves the lowest RMSE and MAE on each dataset.

This cross-market resilience is attributed to the model’s modular design. The EMA block filters out noise and aligns long-term trends across different market regimes, allowing the model to focus on short-term signals with high precision. CBAM attention dynamically reallocates weight across indicators—emphasizing liquidity and momentum metrics in volatile contexts (e.g., Tesla, Moutai, Ping An) and smoothing inter-indicator dependencies in more stable contexts like Amazon and Apple. The gated fusion mechanism adaptively balances the contributions of time-series and image modalities depending on asset-specific market conditions.

Notably, all these results are achieved without any market-specific feature engineering or parameter adjustment, underscoring the model’s generalization strength and plug-and-play potential for cross-border, multi-asset trading environments. The consistent outperformance against strong baselines—including the latest SOTA transformer architectures—further validates TIC-FusionNet as a robust and versatile tool for real-world financial forecasting and risk-sensitive decision-making.

### 7.3 Model interpretability

To address the longstanding “black-box” criticism in deep learning, TIC-FusionNet incorporates a dedicated channel-attention explanation mechanism. By analysing the distribution of learned weights, it is possible to identify which technical indicators dominate under specific market conditions. For example, in downtrends, attention often shifts toward RSI and volume, while in uptrends, moving averages and Bollinger bands become more salient. This interpretability not only boosts model transparency but also enhances investor trust by offering traceable and logical insights into the decision process.

Ablation results indicate that redistributing only the top-5 channel features preserves 93% of the model’s forecasting accuracy, validating the fidelity of attribution. Going forward, interpretability could be further enriched by integrating Grad-CAM to visualise attention distribution over candlestick image inputs, highlighting chart patterns like breakouts or reversals. Additionally, pairing these insights with a natural language explanation module would allow investors to understand model decisions in plain English or Chinese, ultimately supporting real-time explanation dashboards for live trading use. This direction paves the way for a fully explainable and interactive financial AI system.

### 7.4 Real-world application scenarios

The practical implications of TIC-FusionNet extend far beyond academic evaluation. In real-world quantitative trading, the model’s 5-day log-return predictions and attention-derived confidence scores can serve as signals in timing strategies or alpha-enhanced portfolios. For instance, its outputs can trigger breakout-based strategies when the predicted trajectory exceeds recent resistance, or signal mean reversion under declining volatility. For institutional asset managers, the model’s high consistency across market types enables dynamic asset rebalancing—systematically shifting exposure toward assets with superior risk-return projections.

Moreover, TIC-FusionNet’s ability to pre-emptively detect unusual price movements or structural breaks can support risk control functions such as Value-at-Risk (VaR) alarms and regulatory stress-testing. These capabilities are further amplified when the model is embedded into closed-loop decision systems, such as reinforcement learning agents that continuously refine their strategies based on updated forecast states. In summary, TIC-FusionNet is not merely a high-performing model on benchmarks—it is a practical, robust, and interpretable tool tailored for intelligent asset allocation, portfolio optimisation, and real-time financial analytics.

### 7.5 Applicability beyond stock markets

While the present study primarily focuses on stock market forecasting, the proposed TIC-FusionNet framework exhibits substantial potential for application across a wide spectrum of domains in which multimodal temporal dynamics are central to predictive performance. The model’s modular architecture—comprising trend decomposition, modality-specific encoders, and a gated fusion mechanism—provides a generalizable design paradigm for heterogeneous data integration and robust forecasting under nonstationary conditions.

In the **energy systems** domain, high-resolution numerical time series from smart meters or SCADA systems can be fused with geospatial and meteorological satellite imagery to enhance load demand forecasting, renewable generation prediction, and grid stability assessment. The trend decomposition capability of TIC-FusionNet is particularly beneficial for isolating seasonal consumption cycles from short-term fluctuations induced by weather anomalies.

Within **transportation and mobility analytics**, traffic flow measurements and vehicle trajectory time series can be integrated with video streams from roadside surveillance or UAVs to enable more accurate congestion forecasting, incident detection, and dynamic route optimization. Here, the cross-modal attention and gated fusion can dynamically reweight visual and sensor-derived signals as traffic states evolve.

In **environmental monitoring**, pollutant concentration time series can be combined with remote sensing or hyperspectral imagery to forecast air quality indices, detect algal blooms, and track land-use change. The noise-suppression properties of the EMA decomposition block help mitigate measurement artifacts and irregular sampling that are common in environmental datasets.

Finally, in **healthcare and biomedical** applications, physiological signals such as ECG and EEG, as well as continuous glucose monitoring data, can be jointly modeled with imaging modalities (e.g., echocardiograms, MRI, retinal fundus images) for early disease detection, progression tracking, and personalized treatment planning. The interpretability features of TIC-FusionNet can provide clinicians with modality-specific importance scores, facilitating trust and adoption in clinical decision support.

Across these domains, the common challenges—heterogeneous modalities, asynchronous sampling rates, complex interdependencies, and high noise—align closely with the design motivations of TIC-FusionNet. As such, the framework represents a domain-agnostic, extensible solution for multimodal time series forecasting that can support both operational decision-making and long-horizon planning in diverse real-world contexts.

## 8 Conclusion

This paper presents TIC-FusionNet, a trend-aware multimodal neural architecture that achieves state-of-the-art results across six diverse stock datasets, designed to improve the accuracy, robustness, and interpretability of stock price forecasting under nonlinear and nonstationary market conditions. The proposed model systematically integrates three core components: (1) an Exponential Moving Average (EMA) decomposition module for isolating long-term trends and suppressing short-term noise; (2) a dual-branch encoder comprising a Linear Transformer for temporal modeling and a CNN enhanced with Convolutional Block Attention Module (CBAM) for image-based technical pattern recognition; and (3) a gated fusion mechanism that dynamically aligns and aggregates modality-specific embeddings based on market volatility and contextual salience.

Comprehensive empirical evaluations were conducted on six real-world stock datasets spanning multiple market regimes and sectors: Amazon (technology), Apple (consumer electronics), Tesla (high-volatility growth), Moutai (consumer goods), Ping An (finance), and Vanke (real estate). TIC-FusionNet consistently outperforms classical statistical models (Linear Regression, SVR), ensemble learning methods (Random Forest), deep neural networks (LSTM, CNN-only, TCN), and Transformer-based architectures (Informer, Autoformer, Crossformer, and iTransformer), achieving up to 25.4% reduction in RMSE relative to Random Forest, 21.5% versus LSTM, 18.9% versus Autoformer, and 17.3% versus iTransformer, and yielding a highest *R*^2^ score of 0.9264 on Apple and above 0.87 across all datasets. These results highlight the model’s superior generalization capability and volatility resilience across diverse financial contexts.

Ablation studies further validate the architectural design: removing the CBAM module degrades performance by increasing RMSE by 7.0%, while omitting EMA decomposition reduces trend isolation efficacy and lowers *R*^2^ by up to 0.03. The absence of residual connections results in training instability on highly volatile assets such as Tesla and Moutai, confirming the necessity of each module for convergence and predictive reliability.

In terms of interpretability, TIC-FusionNet supports channel-spatial attention inspection, enabling identification of dominant technical features under different market phases (e.g., RSI and volume during downtrends, moving averages in bullish regimes). Case studies on Tesla and Apple show that attention maps capture sector-specific signals such as momentum bursts in growth stocks and supply-chain cycle indicators in consumer electronics. This functionality enhances transparency and facilitates model diagnostics for financial practitioners.

From a deployment perspective, TIC-FusionNet can be integrated into real-time decision support systems. Its five-day log-return predictions and volatility-weighted attention scores may inform breakout detection, mean-reversion strategies, and adaptive portfolio optimization frameworks. The architecture also provides a foundation for early warning systems in risk-sensitive financial applications. With per-sample inference latency of ∼1.08 ms in batch mode and moderate GPU memory usage (1.15 GB), the model is suitable for near-real-time operations in production environments.

Beyond predictive accuracy, we also quantified the economic value of TIC-FusionNet’s forecasts via backtesting a simple long/short strategy across all six datasets. Risk-adjusted performance metrics (Sharpe, Information Ratio, Calmar Ratio, and Maximum Drawdown) consistently favored TIC-FusionNet over all baselines (see Supporting Information S4 Appendix). These results provide empirical evidence that the model’s lower forecast errors translate into superior return profiles with reduced downside risk, reinforcing its practical relevance for real-world trading and portfolio management.

Nevertheless, limitations remain. TIC-FusionNet is currently optimized for daily-level forecasting and may require architectural modifications for high-frequency or intraday prediction. Its dependency on historical time series and visual patterns limits responsiveness to exogenous shocks such as geopolitical crises or macroeconomic policy changes. Additionally, the model does not explicitly encode inter-stock relational dependencies or textual sentiment signals.

Future work will focus on three directions: (1) incorporating Graph Neural Networks (GNNs) to capture structural dependencies among correlated assets and industry sectors; (2) integrating external sentiment sources (e.g., financial news, social media) to enrich contextual awareness; and (3) coupling TIC-FusionNet with reinforcement learning agents for closed-loop trading systems that jointly optimize prediction and action policies. Additional extensions will explore mixed-frequency modeling to unify daily and intraday forecasts, and lightweight variants for edge-device deployment in latency-sensitive trading scenarios. These extensions aim to further enhance the model’s adaptability, interpretability, and real-world utility in intelligent financial systems.

## Supporting information

S1 Appendix Robustness to varying historical data lengths. (PDF)

S2 Appendix Complete Training Settings and Hyperparameters. (PDF)

S3 Appendix Statistical Significance and Robustness Verification. (PDF)

S4 Appendix Return-Oriented Evaluation and Financial Metrics. (PDF)

S5 Appendix Ablation Studies on TIC-FusionNet Components. (PDF)

## References

[1] Box GEP, Jenkins GM, Reinsel GC, Ljung GM. Time series analysis: forecasting and control. John Wiley & Sons; 2015.

[2] Bollerslev T. Generalized autoregressive conditional heteroskedasticity. Journal of Econometrics. 1986;31(3):307–27.

[3] Cont R. Empirical properties of asset returns: stylized facts and statistical issues. Quantitative Finance. 2001;1(2):223.

[4] Sain STR. The nature of statistical learning theory. 1996.

[5] Breiman L. Random forests. Machine learning. 2001;45:5–32.

[6] Friedman JEH. Greedy function approximation: a gradient boosting machine. Annals of Statistics. 2001:1189–232.

[7] Fischer T, Krauss C. Deep learning with long short-term memory networks for financial market predictions. European Journal of Operational Research. 2018;270(2):654–69.

[8] Hoseinzade E, Haratizadeh S. CNNpred: CNN-based stock market prediction using a diverse set of variables. Expert Systems with Applications. 2019;129:273–85.

[9] Vaswani A, Shazeer N, Parmar N, Uszkoreit J, Jones L, Gomez AN, et al. Attention is all you need. Advances in neural information processing systems. 2017;30.

[10] Zhou H, Zhang S, Peng J, Zhang S, Li J, Xiong H, et al. Informer: Beyond efficient transformer for long sequence time-series forecasting. In: Proceedings of the AAAI Conference on Artificial Intelligence. 2021. p. 11106–15.

[11] Haixu W, Jiehui X, Wang J, Mingsheng L. Decomposition transformers with auto-correlation for long-term series forecasting. 2022.

[12] Liu Y, Hu T, Zhang H, Wu H, Wang S, Ma L, et al. Itransformer: Inverted transformers are effective for time series forecasting. 2023. https://arxiv.org/abs/2310.06625

[13] Zhang Y, Yan J. Crossformer: Transformer utilizing cross-dimension dependency for multivariate time series forecasting. 2023.

[14] Wu H, Xu J, Wang J, Long M. Autoformer: decomposition transformers with auto-correlation for long-term series forecasting. Advances in Neural Information Processing Systems. 2021;34:22419–30.

[15] Khodaee P, Esfahanipour A, Taheri HM. Forecasting turning points in stock price by applying a novel hybrid CNN-LSTM-ResNet model fed by 2D segmented images. Engineering Applications of Artificial Intelligence. 2022;116:105464.

[16] He S, Gu S. Multi-modal attention network for stock movements prediction. arXiv preprint. 2021. doi: arXiv:2112.13593

[17] Zong C, Shao J, Lu W, Zhuang Y. Stock movement prediction with multimodal stable fusion via gated cross-attention mechanism. arXiv preprint 2024. doi: arXiv:2406.06594

[18] Lee SI l, Yoo SJ. Multimodal deep learning for finance: integrating and forecasting international stock markets. The Journal of Supercomputing. 2020;76:8294–312.

[19] Radulescu M, Rao A, Dogan B, Abbas S. Co-movement between COVID-19, oil price and American stock market during 2020 : fresh investigation from partial and multiple wavelet methods. Resources Policy. 2024;95:105194.

[20] Gao Y, Zhao C, Wang Y. Investor sentiment and stock returns: new evidence from Chinese carbon-neutral stock markets based on multi-source data. International Review of Economics & Finance. 2024;92:438–50.

[21] Shumway RH, Stoffer DS. ARIMA models. Time series analysis and its applications: with R examples. 2017. p. 75–163.

[22] Sezer OB, Gudelek MU, Ozbayoglu AM. Financial time series forecasting with deep learning: a systematic literature review: 2005 –2019. Applied Soft Computing. 2020;90:106181.

[23] Woo S, Park J, Lee J-Y, Kweon IS. Cbam: convolutional block attention module. In: Proceedings of the European conference on computer vision (ECCV). 2018. p. 3–19.

[24] Zadeh AB, Liang PP, Poria S, Cambria E, Morency L-P. Multimodal language analysis in the wild: CMU-mosei dataset and interpretable dynamic fusion graph. In: Proceedings of the 56th Annual Meeting of the Association for Computational Linguistics (Volume 1: Long Papers). 2018. p. 2236–46.

[25] Zou S, Huang X, Shen X. Multimodal prompt transformer with hybrid contrastive learning for emotion recognition in conversation. In: Proceedings of the 31st ACM International Conference on Multimedia. 2023. p. 5994–6003.

[26] Patel J, Shah S, Thakkar P, Kotecha K. Predicting stock and stock price index movement using trend deterministic data preparation and machine learning techniques. Expert systems with applications. 2015;42(1):259–68.

[27] Ballings M, Van den Poel D, Hespeels N, Gryp R. Evaluating multiple classifiers for stock price direction prediction. Expert Systems with Applications. 2015;42(20).

[28] Wasserbacher H e l m u t, Spindler M a r t i n. Machine learning for financial forecasting, planning and analysis: recent developments and pitfalls. Digital Finance. 2022;4(1):63–88.

[29] Bao W, Yue J, Rao Y. A deep learning framework for financial time series using stacked autoencoders and long-short term memory. PLoS One. 2017;12(7):e0180944. doi: 10.1371/journal.pone.0180944 28708865 PMC5510866

[30] Yu Y, Si X, Hu C, Zhang J. A review of recurrent neural networks: LSTM cells and network architectures. Neural Comput. 2019;31(7):1235–70. doi: 10.1162/neco_a_01199 31113301

[31] Sherstinsky, Alex. Fundamentals of recurrent neural network (RNN) and long short-term memory (LSTM) network. Physica D: Nonlinear Phenomena. 2020;404:132306.

[32] Graves A, Graves A. Long short-term memory. In: Supervised sequence labelling with recurrent neural networks, 2012. p. 37–45.

[33] Nelson DMQ, Pereira ACM, De Oliveira RA. Stock market’s price movement prediction with LSTM neural networks. In: 2017 International Joint Conference on Neural Networks (IJCNN). 2017. p. 1419–26.

[34] Tsai Y-HH, Bai S, Pu Liang P, Kolter JZ, Morency L-P, Salakhutdinov R. Multimodal transformer for unaligned multimodal language sequences. Proc Conf Assoc Comput Linguist Meet. 2019;2019:6558–69. doi: 10.18653/v1/p19-1656 32362720 PMC7195022

[35] Jiang Y, Ning K, Pan Z, Shen X, Ni J, Yu W, et al. Multi-modal time series analysis: A tutorial and survey. In: Proceedings of the 31st ACM SIGKDD Conference on Knowledge Discovery and Data Mining, 2025. p. 6043–53.

[36] Zong C, Wan J, Cascone L, Zhou H. Stock movement prediction with multimodal stable fusion via gated cross-attention mechanism. Complex & Intelligent Systems. 2025;11(9):396.

[37] Marino JA, Arrieta-Prieto ME, CalderÂ´on VSA. Comparison between statistical models and machine learning for forecasting multivariate time series: An empirical approach. Communications in Statistics: Case Studies, Data Analysis and Applications. 2025;11(1):56–91.

[38] Pei Y, Cartlidge J, Mandal A, Gold D, Marcilio E, Mazzon R. Cross-modal temporal fusion for financial market forecasting. arXiv preprint 2025. https://arxiv.org/abs/2504.13522

[39] Gangwani P, Panthi V. Leveraging multimodal data and deep learning for enhanced stock market prediction. AI-Based Advanced Optimization Techniques for Edge Computing. Wiley Online Library; 2025. p. 93–127.

[40] Xing Y, Yan C, Xie CC. Predicting NVIDIA’s next-day stock price: A comparative analysis of LSTM, MLP, ARIMA, and ARIMA-GARCH models. In: World Congress in Computer Science, Computer Engineering & Applied Computing. 2024. p. 467–79.

[41] Chowdhury MS, Nabi N, Rana MNU, Shaima M, Esa H, Mitra A, et al. Deep learning models for stock market forecasting: a comprehensive comparative analysis. Journal of Business and Management Studies. 2024;6(2):95.

[42] Farhadi A, Zamanifar A, Alipour A, Taheri A, Asadolahi M. A hybrid LSTM-GRU model for stock price prediction. IEEE Access. 2025;13.

[43] Ali Z. A comprehensive overview and comparative analysis of CNN, RNN-LSTM and Transformer. 2024.

[44] Biswas AK, Bhuiyan MS, Mir MNH, Rahman A, Mridha MF, Islam MR, et al. A dual output temporal convolutional network with attention architecture for stock price prediction and risk assessment. IEEE Access. 2025.

[45] Li N, Chao G, Zou J, Jiang G. SPPMFN: efficient multimodal financial time-series prediction network with self-supervised learning. IEEE Access. 2025.

