# Supplementary material for: TIC-FusionNet: A multimodal deep learning framework with temporal decomposition and attention-based fusion for time series forecasting
Source: PLoS One. 2025 Oct 9;20(10):e0333379. doi: 10.1371/journal.pone.0333379 (PMC12510716; doi:10.1371/journal.pone.0333379)
Supplement: S1 Appendix — (PDF) [file pone.0333379.s001.pdf]

Supporting Information

S1 Robustness to Varying Historical Data Lengths

To examine the robustness of TIC-FusionNet under different historical data horizons, supplementary experiments were conducted by resampling the Chinese stock datasets (*Moutai*, *Ping An*, *Vanke*) to 6,155 trading days and trimming the foreign stock datasets (*Amazon*, *Apple*, *Tesla*) to 1,500 trading days. This setting simulates both extended and shortened input scenarios. All experimental settings (feature construction, splits, and hyperparameters) were kept consistent with the main evaluation, except for the modified sequence lengths.

Table 1: *

S1 Table. Performance with extended historical horizon (6,155 trading days) on Chinese stocks. Best results are in **bold**.

Model	RMSE	MAE	R^2	SMAPE
LR	0.0750	0.0570	0.8520	12.14
SVR	0.0735	0.0562	0.8564	11.88
RF	0.0712	0.0543	0.8642	11.52
LSTM	0.0695	0.0528	0.8684	10.97
Informer	0.0688	0.0521	0.8701	10.83
Autoformer	0.0682	0.0517	0.8712	10.71
Crossformer	0.0679	0.0514	0.8723	10.68
iTransformer	0.0675	0.0512	0.8731	10.64
CNN-only	0.0708	0.0539	0.8653	11.48
TCN	0.0692	0.0525	0.8695	10.92
**Ours**	**0.0629**	**0.0483**	**0.8857**	**9.87**

Table 2: *

S2 Table. Performance with shortened historical horizon (1,500 trading days) on foreign stocks. Best results are in **bold**.

Model	RMSE	MAE	R^2	SMAPE
LR	0.0720	0.0548	0.8610	11.65
SVR	0.0705	0.0539	0.8653	11.42
RF	0.0683	0.0512	0.8751	10.84
LSTM	0.0667	0.0504	0.8795	10.42
Informer	0.0659	0.0499	0.8812	10.35
Autoformer	0.0655	0.0497	0.8820	10.29
Crossformer	0.0652	0.0495	0.8828	10.23
iTransformer	0.0649	0.0493	0.8836	10.18
CNN-only	0.0675	0.0509	0.8774	10.61
TCN	0.0663	0.0502	0.8803	10.38
**Ours**	**0.0608**	**0.0471**	**0.8924**	**9.72**

The results confirm that TIC-FusionNet maintains a clear performance margin under both extended and shortened data conditions, indicating strong robustness and adaptability.
