# Supplementary material for: TIC-FusionNet: A multimodal deep learning framework with temporal decomposition and attention-based fusion for time series forecasting
Source: PLoS One. 2025 Oct 9;20(10):e0333379. doi: 10.1371/journal.pone.0333379 (PMC12510716; doi:10.1371/journal.pone.0333379)
Supplement: S2 Appendix — (PDF) [file pone.0333379.s002.pdf]

Supporting Information

S2 Complete Training Settings and Hyperparameters

This appendix reports the full training settings, search ranges, and selected hyperparameters for all models evaluated in the paper. Unless otherwise stated, experiments were conducted with PyTorch 1.10 on a single NVIDIA Tesla V100 (32 GB), using Adam optimizer, MSE loss, early stopping (patience=10), and ReduceLROnPlateau scheduling.

Table 1: *
S3 Table. Common data and training setup.

**Item**	**Setting**
Window length T	30 trading days (sliding window)
Prediction horizon h	5 trading days (log-return target)
Normalization	Numerical series: MinMax; technical indicators: Z-score; images normalized to [0,1] then $(x - 0.5)/0.5$
Image resolution	160×120 RGB candlestick charts with SMA/BB/RSI/Volume overlays
Train/Val/Test	Chronological split 70/10/20 (no shuffling)
Optimizer / Loss	Adam, MSE loss
LR schedule	ReduceLROnPlateau (factor 0.5, patience 3, min LR 10^{-6})
Batch size / Epochs	256 (numerical), 128 (image or multimodal); max 100 epochs; early stopping patience=10
Seeds / Repeats	3 random seeds; mean±std reported
Hardware / Software	Tesla V100 (32 GB), PyTorch 1.10, Python 3.8
