# Supplementary material for: TIC-FusionNet: A multimodal deep learning framework with temporal decomposition and attention-based fusion for time series forecasting
Source: PLoS One. 2025 Oct 9;20(10):e0333379. doi: 10.1371/journal.pone.0333379 (PMC12510716; doi:10.1371/journal.pone.0333379)
Supplement: S3 Appendix — (PDF) [file pone.0333379.s003.pdf]

Supporting Information

S3 Statistical Significance and Robustness Verification

Paired statistical significance tests (paired t-test and Wilcoxon signed-rank test) were conducted for RMSE, MAE, SMAPE, and R^2. Values below 0.05 indicate statistically significant improvements of TIC-FusionNet over baselines.

Table 1: *
S4 Table. Statistical significance test results (p-values) comparing TIC-FusionNet with each baseline. Bold indicates $p < 0.05$.

**Model**	**RMSE**	**MAE**	**SMAPE**	R^2
LR	**0.013 / 0.017**	**0.016 / 0.020**	**0.014 / 0.018**	**0.021 / 0.026**
SVR	**0.015 / 0.019**	**0.018 / 0.023**	**0.016 / 0.021**	**0.025 / 0.031**
RF	**0.012 / 0.016**	**0.015 / 0.019**	**0.013 / 0.017**	**0.020 / 0.025**
LSTM	**0.021 / 0.034**	**0.024 / 0.036**	**0.022 / 0.034**	**0.028 / 0.040**
Informer	**0.009 / 0.011**	**0.012 / 0.015**	**0.011 / 0.014**	**0.017 / 0.021**
Autoformer	0.063 / 0.072	0.071 / 0.082	0.068 / 0.079	0.075 / 0.087
Crossformer	**0.018 / 0.025**	**0.021 / 0.029**	**0.019 / 0.027**	**0.026 / 0.034**
iTransformer	**0.032 / 0.051**	**0.036 / 0.056**	**0.034 / 0.054**	**0.040 / 0.060**
CNN-only	**0.015 / 0.028**	**0.019 / 0.031**	**0.017 / 0.029**	**0.023 / 0.036**
TCN	**0.027 / 0.037**	**0.030 / 0.042**	**0.029 / 0.040**	**0.034 / 0.046**
