# Supplementary material for: TIC-FusionNet: A multimodal deep learning framework with temporal decomposition and attention-based fusion for time series forecasting
Source: PLoS One. 2025 Oct 9;20(10):e0333379. doi: 10.1371/journal.pone.0333379 (PMC12510716; doi:10.1371/journal.pone.0333379)
Supplement: S4 Appendix — (PDF) [file pone.0333379.s004.pdf]

Supporting Information

S4 Return-Oriented Evaluation and Financial Metrics

A daily-rebalanced long/short strategy was backtested using the 5-day log-return forecast. To evaluate risk-adjusted investment performance, we adopt four widely used financial metrics: Sharpe ratio, Maximum Drawdown (MDD), Information Ratio (IR), and Calmar ratio. Their mathematical definitions are as follows:

$$\text{Sharpe Ratio} = \frac{E[R_p - R_f]}{\sigma_p}, \tag{1}$$

$$\text{MDD} = \min_t \left(\frac{V_t - \max_{s \le t} V_s}{\max_{s \le t} V_s} \right), \tag{2}$$

$$\text{Information Ratio (IR)} = \frac{E[R_p - R_b]}{\sigma_{p-b}}, \tag{3}$$

$$\text{Calmar Ratio} = \frac{\text{Annualized Return}}{|\text{MDD}|}, \tag{4}$$

where R_p is the portfolio return, R_f is the risk-free rate, σ_p is the standard deviation of portfolio returns, V_t is the portfolio value at time t, R_b is the benchmark return, and σ_{p-b} is the tracking error relative to the benchmark.

Table 1: *
S5 Table. Risk-adjusted performance (mean across datasets; best in **bold**).

**Model**	**Sharpe**	**MDD (%)**	**Information Ratio**	**Calmar**
LR	0.85	-21.3	0.72	0.41
SVR	0.88	-20.7	0.74	0.43
RF	0.92	-19.9	0.78	0.46
LSTM	0.97	-19.4	0.82	0.50
Informer	1.01	-18.7	0.85	0.54
Autoformer	1.03	-18.1	0.86	0.57
Crossformer	1.05	-17.9	0.88	0.59
iTransformer	1.06	-17.6	0.89	0.60
CNN-only	0.89	-20.4	0.76	0.44
TCN	0.96	-19.1	0.81	0.51
**Ours**	**1.18**	**-16.2**	**0.94**	**0.67**

Results indicate that TIC-FusionNet attains the highest risk-adjusted return (Sharpe/IR) and the smallest drawdown, aligning with its lower forecasting errors in the main tables.
