# Supplementary material for: TIC-FusionNet: A multimodal deep learning framework with temporal decomposition and attention-based fusion for time series forecasting
Source: PLoS One. 2025 Oct 9;20(10):e0333379. doi: 10.1371/journal.pone.0333379 (PMC12510716; doi:10.1371/journal.pone.0333379)
Supplement: S5 Appendix — (PDF) [file pone.0333379.s005.pdf]

Supporting Information

S5 Result of Ablation Studies

Table S1: *

S6 Table. Ablation results (RMSE/MAE/R^2/MAPE/SMAPE) for the Amazon dataset. Mean±std over three seeds. Best results are **bold**.

**Variant**	RMSE	MAE	R^2	MAPE	SMAPE
**TIC-FusionNet (ours)**	**0.0534**	**0.0427**	**0.8737**	**8.73**	**5.42**
w/o Image	0.0587	0.0478	0.8213	9.43	5.75
w/o EMA	0.0555	0.0448	0.8606	9.08	5.58
w/o Attention	0.0566	0.0461	0.8475	9.17	5.64

Table S2: *

S7 Table. Ablation results (RMSE/MAE/R^2/MAPE/SMAPE) for the Apple dataset (simulated).

**Variant**	RMSE	MAE	R^2	MAPE	SMAPE
**TIC-FusionNet (ours)**	**0.0542**	**0.0412**	**0.8895**	**7.85**	**5.48**
w/o Image	0.0599	0.0461	0.8532	8.44	5.89
w/o EMA	0.0567	0.0439	0.8718	8.11	5.65
w/o Attention	0.0576	0.0448	0.8642	8.23	5.72

Table S3: *

S8 Table. Ablation results (RMSE/MAE/R^2/MAPE/SMAPE) for the Tesla dataset (simulated).

**Variant**	RMSE	MAE	R^2	MAPE	SMAPE
**TIC-FusionNet (ours)**	**0.0612**	**0.0478**	**0.9021**	**7.35**	**5.89**
w/o Image	0.0675	0.0532	0.8703	8.19	6.34
w/o EMA	0.0643	0.0507	0.8841	7.82	6.12
w/o Attention	0.0651	0.0516	0.8799	7.95	6.18

Table S4: *

S9 Table. Ablation results (RMSE/MAE/R^2/MAPE/SMAPE) for the Moutai dataset.

**Variant**	RMSE	MAE	R^2	MAPE	SMAPE
**TIC-FusionNet (ours)**	**0.0587**	**0.0463**	**0.9155**	**6.97**	**6.04**
w/o Image	0.0712	0.0509	0.8348	8.62	7.12
w/o EMA	0.0654	0.0481	0.8702	7.99	6.64
w/o Attention	0.0631	0.0499	0.8872	7.86	6.36

Table S5: *

S10 Table. Ablation results (RMSE/MAE/R^2/MAPE/SMAPE) for the Ping An dataset.

**Variant**	RMSE	MAE	R^2	MAPE	SMAPE
**TIC-FusionNet (ours)**	**0.0576**	**0.0431**	**0.9099**	**6.77**	**5.69**
w/o Image	0.0622	0.0481	0.8629	7.93	6.34
w/o EMA	0.0598	0.0452	0.8914	7.25	6.20
w/o Attention	0.0605	0.0462	0.8861	7.54	6.09

Table S6: *

S11 Table. Ablation results (RMSE/MAE/R^2/MAPE/SMAPE) for the Vanke dataset.

**Variant**	RMSE	MAE	R^2	MAPE	SMAPE
**TIC-FusionNet (ours)**	**0.0574**	**0.0433**	**0.8706**	**6.57**	**7.05**
w/o Image	0.0619	0.0480	0.8283	7.64	7.55
w/o EMA	0.0601	0.0456	0.8504	7.21	7.31
w/o Attention	0.0603	0.0462	0.8451	7.32	7.22
